# Distribution of Woody Plant Species Among Different Disturbance Regimes of Forests in a Temperate Deciduous Broad-Leaved Forest

**DOI:** 10.3389/fpls.2021.618524

**Published:** 2021-04-06

**Authors:** Jingjing Xi, Yizhen Shao, Zihao Li, Pengfei Zhao, Yongzhong Ye, Wang Li, Yun Chen, Zhiliang Yuan

**Affiliations:** ^1^College of Life Sciences, Henan Agricultural University, Zhengzhou, China; ^2^Section for Ecoinformatics and Biodiversity, Department of Biology, Aarhus University, Aarhus, Denmark; ^3^State Key Laboratory of Remote Sensing Science, Aerospace Information Research Institute, Chinese Academy of Sciences, Beijing, China; ^4^Institute of Botany, Chinese Academy of Sciences, Beijing, China; ^5^Field Scientific Observation and Research Station of Forest Ecosystem in the North-South Transition Zone of Funiu Mountain, Zhengzhou, China

**Keywords:** disturbance regime, species diversity, forest community, habitat preferences, ecological specialization

## Abstract

Forests in different disturbance regimes provide diverse microhabitats for species growth. However, whether the species distribution of wood plant is random or follows ecological specialization among forests in different disturbance regimes remains to be elucidated. In this study, four 1 hm^2^ (100 m × 100 m) forest dynamic monitoring plots in different disturbance regimes of forests were randomly selected in a temperate deciduous broad-leaved forest. We examined the specificity of woody plants to forests through network analysis. Torus-translation test was used to analyze the species distribution preference of woody plants to forests in different disturbance regimes. The specialization index of woody plants was 0.3126, and that of shrubs (51.01%) was higher than that of trees (25.16%). Moreover, 66.67% (38/57) of woody plants were associated with different forests. More shrub species (70.00%) had specific preferences than tree species (45.95%) with respect to forests in different disturbance regimes. Our findings suggest that the distribution of woody plants among forests with different disturbance regimes is not random but is specialized. Different woody plants show different community preferences in different disturbance regimes of forests. Shrubs show higher specialization than trees in different disturbance regimes of forests.

## Introduction

Disturbance is an important factor that affects spatial heterogeneity and community succession in forest ecosystem (Zhang and Shangguan, 2006). This factor plays an important role in changes in species composition during plant community renewal and reconstruction. In addition to transformations of ecosystems caused by natural disasters, forest communities worldwide are being increasingly altered by human interference (Dong et al., 2005). Human disturbance can have an impact on the biodiversity of forest communities (Singh, 1998; Laurance and Peres, 2006; Laurance et al., 2014). With the intensification of human activities, the influence of human disturbance on ecosystem has gained increasing attention (Ye, 2000; Liu et al., 2002). Studying the effects of human disturbance on species diversity is of great significance for renewal and development of forest communities (Fang et al., 2019).

Forest cutting is a common type of human disturbance. Different cutting methods, intensities, and intervals have different impacts on the structure, function, and biodiversity of forest ecosystem (Chazdon, 2003). Under different human disturbances, environmental differences exist among communities in different succession stages. Different communities differ greatly in biological environments (e.g., community structure, species composition) and abiotic environments (e.g., soil and light) (Yuan et al., 2012; Song et al., 2015; Han et al., 2017; Gallé et al., 2018; Jia et al., 2019). In recent years, many works have reported on the impact of human disturbance and community succession on species diversity (Lu et al., 2008; Lü et al., 2017; Backer et al., 2014; Fang et al., 2019). However, the distribution of woody plants among forests in different disturbance regimes remains unclear.

Ecological specialization is the process by which a species adapts to its living environment and persists in that environment (Devictor et al., 2010; Poisot et al., 2011). In forest community, most species have good environmental adaptability and stable growth and reproduction (Bevill and Louda, 1999). Some species can survive and reproduce under very strict environmental conditions (Barlow et al., 2010). Some species may be able to live in more than one forest but may be better able to live and reproduce in a particular forest. Whether the distribution of species is random or follows ecological specialization among forests of different disturbance regimes remains to be elucidated.

Trees and shrubs belong to two life forms of woody plants (Chen et al., 2014). Many studies have confirmed that their species diversity maintenance mechanisms are different (Debski et al., 2002; Potts et al., 2002; Hao et al., 2014; Chen, 2015). Shrubs are not only affected by topography, light, and soil but also by tree layers to a large extent (Lhotka and Loewenstein, 2008; Parkke and Det, 2008; Hu et al., 2019). However, whether ecological specialization differs for trees and shrubs is not well-understood.

In this study, four 1 hm^2^ (100 m × 100 m) forest dynamic monitoring plots in different disturbance regimes were randomly selected in a temperate deciduous broad-leaved forest. We examined the specificity of woody plants to forests in different disturbance regimes at community and species levels through network analysis and torus-translation, respectively. This study aims to: (1) identify whether the species distribution of woody plants is random or follows ecological specialization among forests of different disturbance regimes; and (2) assess differences in the ecological specialization between trees and shrubs in the examined temperate mountain forest.

## Materials and Methods

### Study Site and Sampling

The Baiyunshan Nature Reserve, which is about 168 km^2^, is located in the south of Henan Province, China (111°48′-112°16′ E, 33°33′-33°56′ N) (Wang et al., 2018) and 1,500–2,216 m above sea level. The slope of the mountain is mostly 40°-80°. The annual precipitation is 1,200 mm, the average annual relative humidity is 70–78%, and the annual average temperature is 18°C. The average temperature is 13.5°C (Bi et al., 2014).

The Baiyunshan Nature Reserve has a transitional climate between subtropical and warm temperate zones and has deciduous broad-leaved forests. The forest coverage in the reserve reaches 98.5%, and it consists of 1,991 species of plants, such as *Quercus aliena* var. *acutiserrata, Carpinus turczaninowii, Betula platyphylla, Pinus armandii Franch*, and *Toxicodendron vernicifluum* (Li et al., 2017).

**Figure 1:**
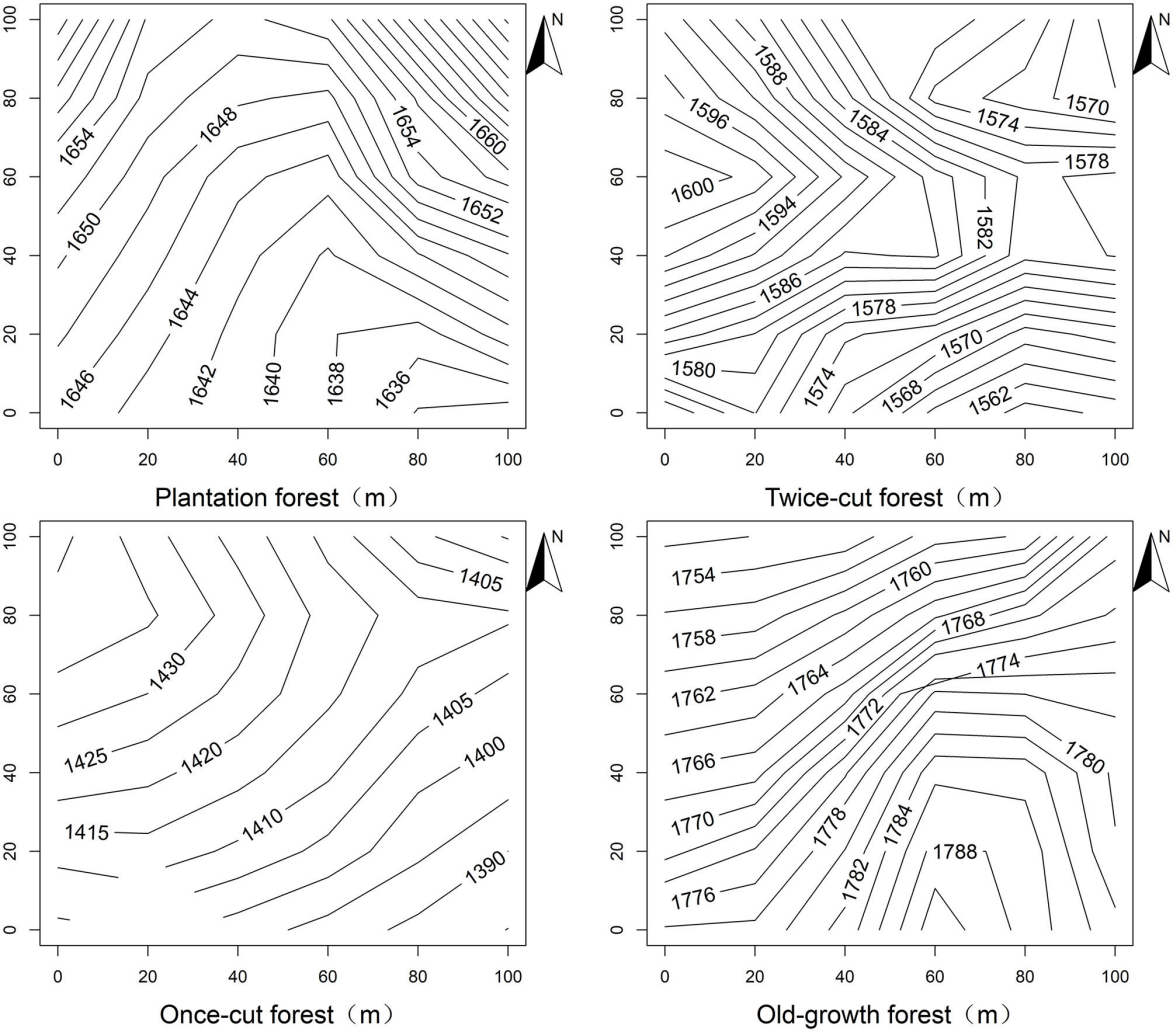
Contour topography map of Baiyunshan plot.

Forest monitoring plots were randomly selected and stratified by disturbance regimes in the Baiyunshan Nature Reserve. Four disturbance regimes of the forest were estimated based on knowledge of local logging events and forest physiognomy. Four 1 hm^2^ plots (100 m × 100 m), namely, plantation forests, twice-cut forests, once-cut forests, and old-growth forests, were randomly selected within each disturbance regime in the reserve (Figure 1). Four 1 hm^2^ plots were divided into 100 grids (10 m × 10 m). All trees with diameter at breast height (DBH) of ≥1 cm in the plot were tagged, mapped, and measured (Condit, 1995). Topographic variables (elevation, convex concave, slope, and aspect) were measured using the method in the study of Harms et al. (2001) for each 10 m × 10 m grid in the plot.

- Plantation forest: It is a *Larix kaempferi* forest planted after logging and clearing and is about 20 years old (high disturbance). The sample plot has 42 species of woody plants, with 31 trees and 11 shrubs. *Quercus aliena* var. *acutiserrata* and *Larix gmelinii* are the dominant species in the field (Table 1 and Supplementary Table 1).
- Twice-cut forest: In this forest, natural regeneration occurred after once-cutting. Twice-cutting and breeding were carried out when the natural recovery was about 30 years old, and natural recovery was carried out, with a stand age of about 50 years (moderate disturbance). The sample plot has 46 species of woody plants, with 31 trees and 15 shrubs. *Quercus aliena* var. *acutiserrata, Pinus armandii Franch*, and *Corylus heterophylla* are the main species in the sample community (Table 1 and Supplementary Table 2).
- Once-cut forest: The forest was restored after comprehensive once-cutting, with a stand age of about 50 years (slight disturbance). The sample plot has 57 species of woody plants, with 43 trees and 14 shrubs. *Quercus aliena* var. *acutiserrata, Pinus armandii Franch*, and *Forsythia suspensa* are the main species of the site (Table 1 and Supplementary Table 3).
- Old-growth forest: In this forest, the individual density, mean DBH, and aboveground biomass of the woody plants were higher than those in the above three plant community types (Burrascano et al., 2013). The forest has been a natural forest for more than 100 years without human disturbance (undisturbance). The sample plot has 52 species of woody plants, with 31 trees and 21 shrubs. *Quercus aliena* var. *acutiserrata, Sorbus hupehensis*, and *Litsea tsinlingensis* are the main species in the sample land (Table 1 and Supplementary Table 4).

**Table 1:** Dominant species in the four communities.

**Species**	**Life form**	**Abundance**	**Mean DBH**	**Basal area at breast height (cm^**2**^)**	**Importance value**
**Plantation forest**					
*Quercus aliena* var. *acutiserrata*	Tree	540.00	13.09	11.30	34.13
*Larix gmelinii*	Tree	125.00	24.26	6.53	14.52
*Pinus tabuliformis Carrière*	Tree	88.00	14.05	1.76	7.16
*Toxicodendron vernicifluum*	Tree	76.00	12.81	1.29	6.83
*Juglans cathayensis*	Tree	45.00	21.55	2.02	6.17
*Cerasus clarofolia*	Tree	28.00	6.75	0.15	2.13
**Twice-cut forest**					
*Quercus aliena* var. *acutiserrata*	Tree	1002.00	10.02	16.30	34.07
*Pinus armandii Franch*	Tree	203.00	14.46	4.01	9.21
*Corylus heterophylla*	Tree	456.00	3.027	0.46	7.66
*Toxicodendron vernicifluum*	Tree	159.00	9.50	1.68	6.27
*Lindera obtusiloba Blume*	Tree	170.00	6.07	0.59	5.03
*Betula platyphylla*	Tree	54.00	11.22	0.86	3.95
**Once-cut forest**					
*Quercus aliena* var. *acutiserrata*	Tree	752.00	14.30	14.90	21.49
*Pinus armandii Franch*	Tree	574.00	11.35	6.63	12.54
*Forsythia suspensa*	Shurb	986.00	2.19	0.69	10.29
*Pinus tabuliformis Carrière*	Tree	343.00	12.57	5.12	9.29
*Quercus serrata* var. *brevipetiolata*	Tree	379.00	8.59	3.67	7.59
*Lindera obtusiloba Blume*	Tree	246.00	4.55	0.55	4.35
**Old-growth forest**					
*Quercus aliena* var. *acutiserrata*	Tree	938.00	16.32	26.39	42.35
*Sorbus hupehensis*	Shurb	382.00	2.71	0.36	6.59
*Litsea tsinlingensis*	Tree	183.00	4.68	0.50	5.05
*Pinus armandii Franch*	Tree	76.00	10.04	1.00	3.88
*Malus honanensis Rehder*	Shurb	104.00	5.09	0.42	3.42
*Salix chaenomeloides Kimura*	Tree	84.00	10.56	0.94	2.94

*Important value was calculated as follows: important value = (relative abundance (%) + relative frequency (%) + relative breast height sectional area (%)/3*.

### Data Analysis

The dominant species in the plots were statistically analyzed based on importance values (IV) to understand the woody plant community structure of the four forests of different disturbance regimes. Important value was calculated as follows: IV = (relative abundance (%) + relative frequency (%) + relative breast height sectional area (%)/3 (Chen et al., 2020). Species accumulation curves of wood plants were drawn using “specaccum” function in vegan package of R to detect diversity difference among the four forests. Differences in the richness and abundance of woody plants in the four communities were statistically analyzed using Kruskal–Wallis method. Betadisper test was conducted with the “vegan” package of R to determine the effects of forests of different disturbance regimes on woody plants (Oksanen et al., 2007; Chen et al., 2020).

In addition to disturbance factors, environmental factors, and interspecific relationships affect species distribution. Therefore, the effects of topographic factors on species distribution as well as interspecific relationships were considered. Redundancy analysis (RDA) was used to evaluate the difference in topographic factors among different communities. Topographic factors include slope, aspect, elevation, and convex concave (Chen et al., 2020). RDA was completed with the “ggord” package of R (Pierre, 2007). Point pattern method was used to analyze the interspecific relations of different species, and the selected function was g(r) function. The 99% confidence interval was simulated by Monte-Carlo (Fan and Yu, 2016). Point pattern was calculated using the “spatstat” package of R.

The relationship between woody plants and the community in the plot was visualized based on network approach to detect the specificity of woody plants to different forests at the community level. The “bipartite” package of R was used to construct a network structure of woody plants and different disturbance regimes of forests (Dormann et al., 2009). We used the H2′ metric of specialization and connectance index to evaluate the relationship between woody plants and different disturbance regimes of forests (Blüthgen et al., 2007; Chen et al., 2020).

Species composition and species distribution preference in the four forests were analyzed by indicator species analysis and torus-translation test, respectively, to detect specificity of woody plants to different disturbance regimes of forests at the species level. “Indicspecies” package of R was used for Indicator species analysis (De Cáceres, 2013). The dependent variable in the indicator species analysis was the species abundance matrix of woody plants. Torus-translation test is currently the most commonly used method for determining the association between species and habitat (Harms et al., 2001; Debski et al., 2002; DeWalt et al., 2006; Gunatilleke et al., 2006; Yamada et al., 2006; Comita et al., 2007). In the present study, four 1 hm^2^ plots with different disturbance regimes of forests were selected as four microhabitats. Based on the 10 m × 10 m subplot in the four 1 hm^2^ plots, the torus-translation test provided 1 real and 1,599 translated maps. Community associations were only tested for species with more than four individuals in the 4 hm^2^ plot. A total of 57 species were used for torus-translation analysis. Further details on this method are provided by Harms et al. (2001). All data were computed in R 3.5.3.

## Results

### Species Composition of Different Disturbance Regimes of Forests

**Figure 2:**
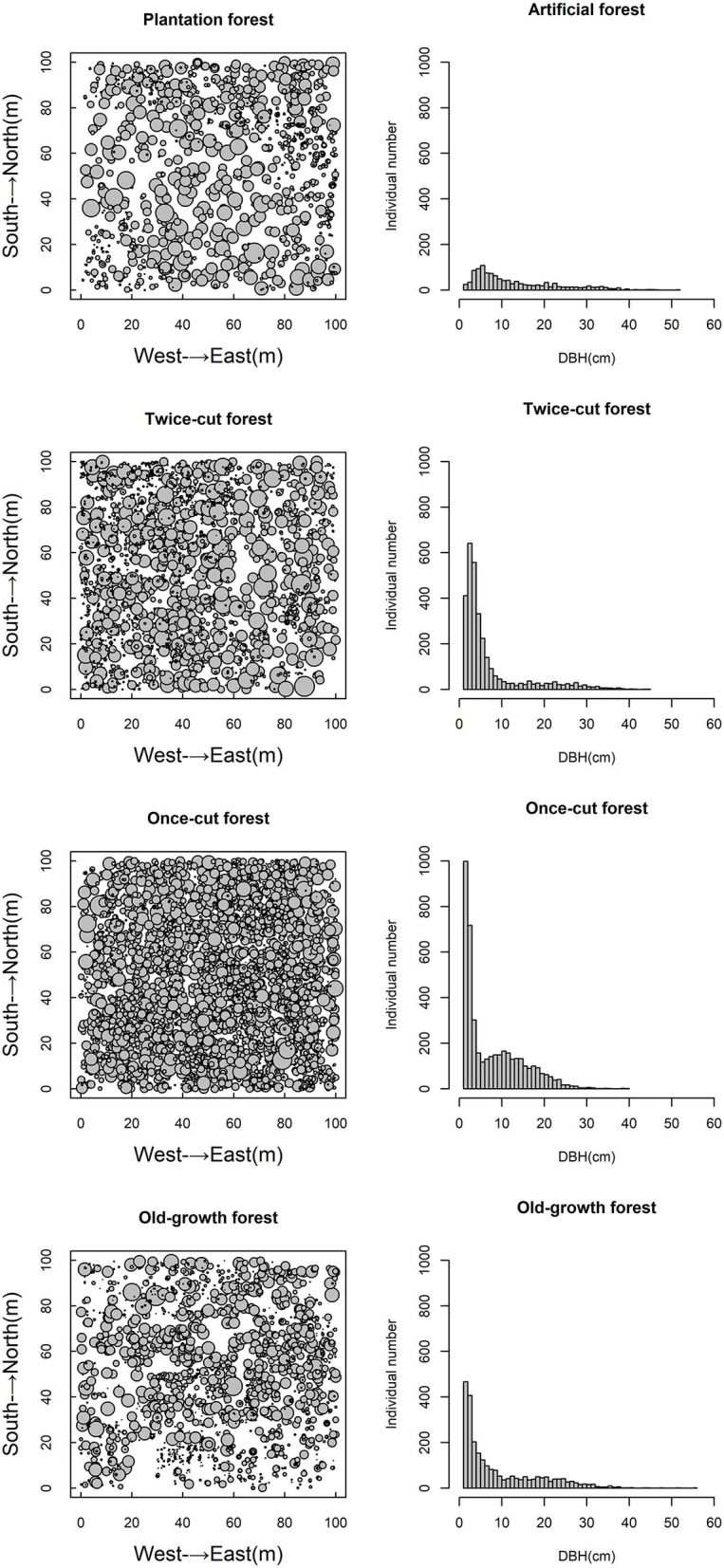
Spatial scatter plots and DBH plots of woody plants in different communities.

**Figure 3:**
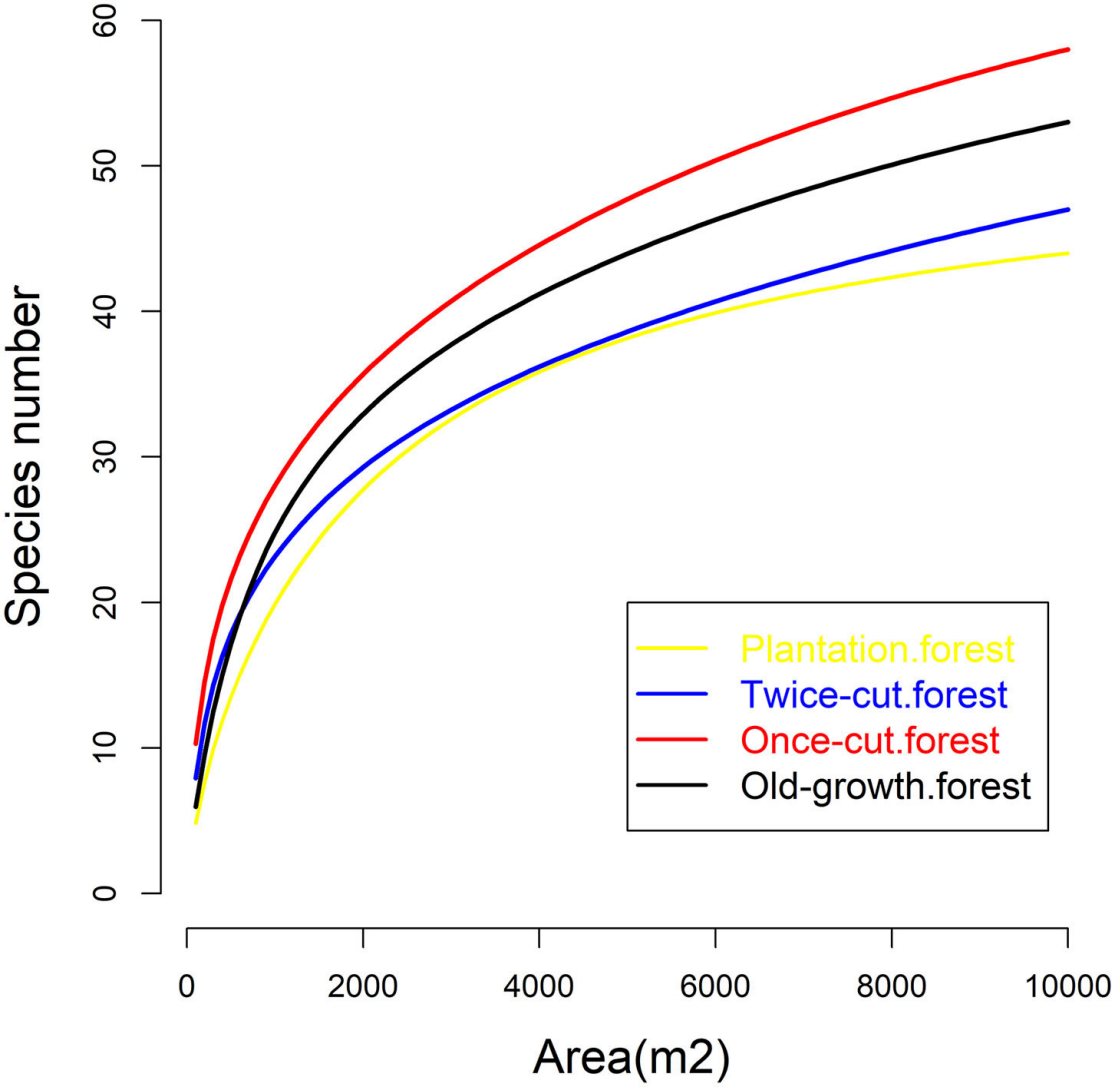
Species area curves of the four communities.

The four communities consisted of 89 species (58 trees and 31 shrubs) of woody plants. The spatial distribution of species among the four communities was highly asymmetric in terms of species density (Figure 2). Once-cut forests had the highest species density, with 4,302 woody plants, followed by twice-cut forests (3,065 woody plants) and old-growth forests (2,490 woody plants). Plantation forests had the lowest species density, with only 1,165 woody plants. The species accumulation curves of the four communities showed that the species number varied with the sampling area (Figure 3).

**Figure 4:**
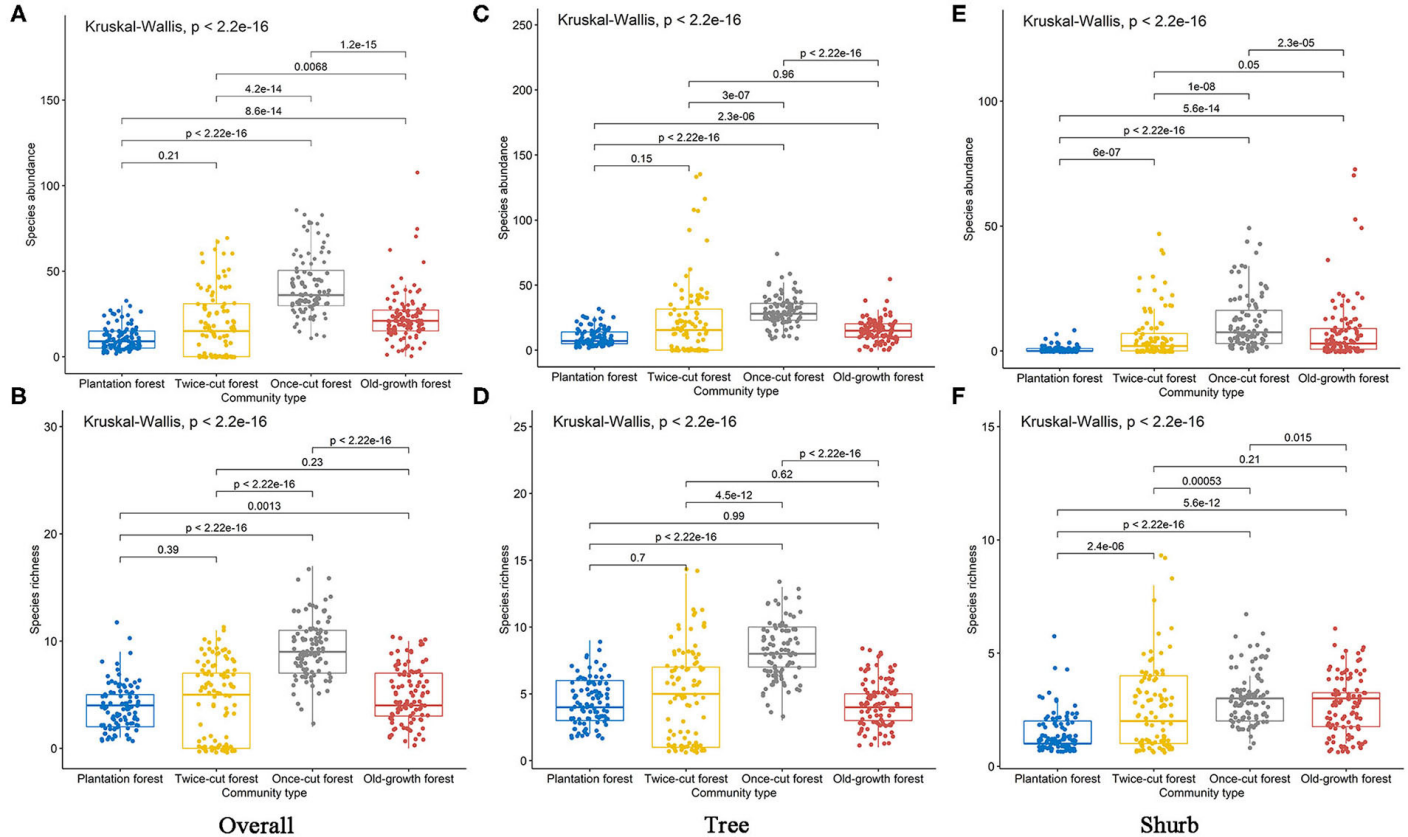
Species richness and abundance of woody plants in the four communities. Black lines indicate significant differences, as obtained using Kruskal–Wallis method (*p* ≤ 0.05 level of significance). **(A,B)** are the abundance and richness of overall species, respectively. **(C,D)** are the abundance and richness of trees, respectively. **(E,F)** are the abundance and richness of shurbs, respectively.

**Figure 5:**
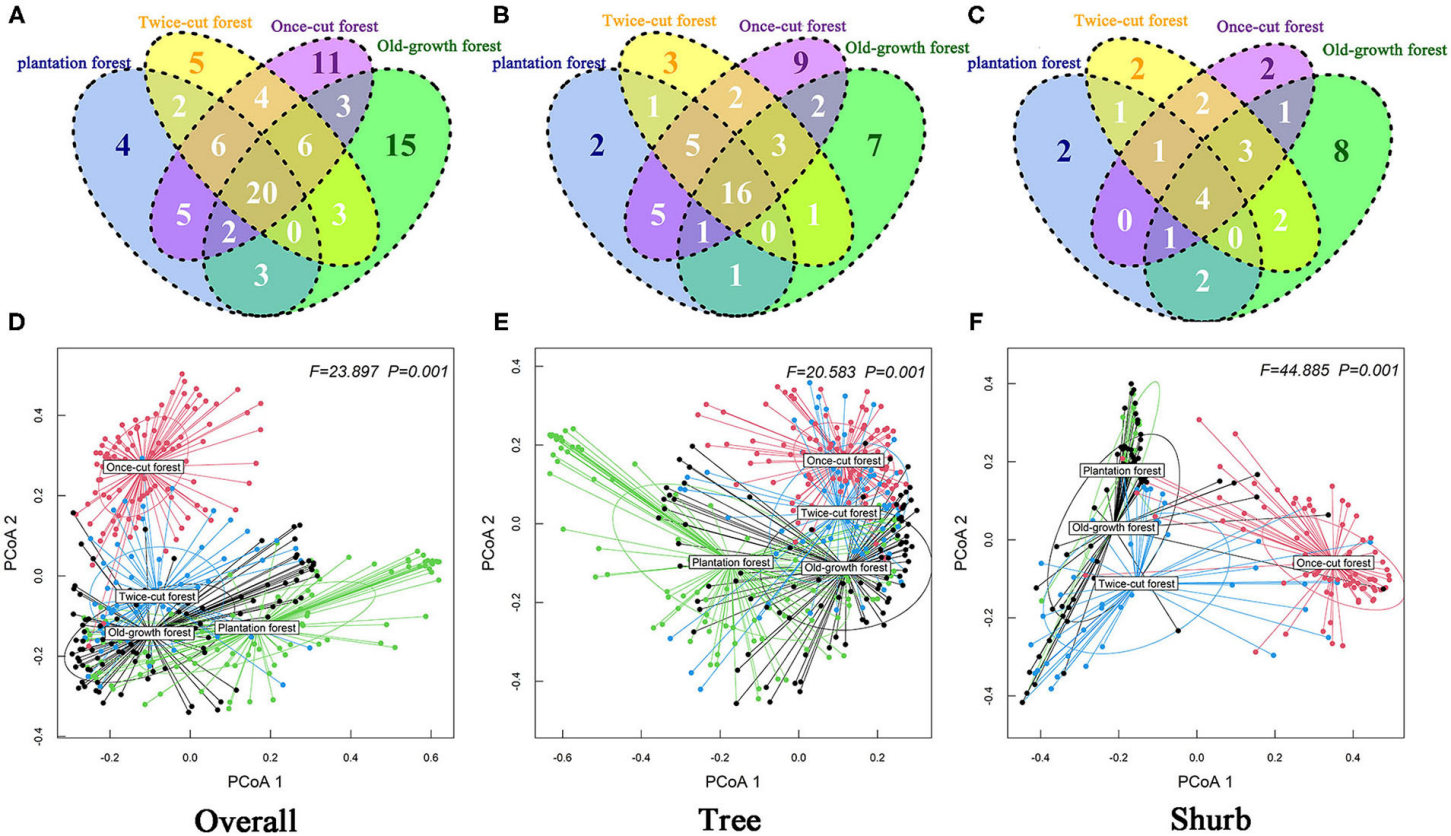
Degree of overlap of the species composition in the four communities (**A**: Overall; **B**: Tree; **C**: Shurb). Effect of community types on beta diversity of the woody plant by running the betadisper function. ANOVA was conducted to test how these distances differ among community (**D**: Overall; **E**: Tree; **F**: Shurb). PCoA1 and PCoA2 are the first and second sort axes in the “betadisper” analysis, respectively.

The Kruskal–Wallis test results showed significant differences in species abundance and species richness among the four communities (Figures 4A–F). The betadisper analysis followed by ANOVA indicated significant differences in the distribution of woody plants among the four communities (Figures 5D–F).

In RDA, topographic factors, namely, elevation, slope, aspect, and convex concave, explained 18.72, 15.45, 11.83, and 9.10% of the variation in the species distribution of the four communities, respectively. The significance of each environmental factor is shown in Supplementary Figure 1. According to the interspecific correlation analysis, most of the dominant species had a significant negative correlation in the four communities at the whole research scale (Supplementary Figures 2–5).

### Species Specialization Characteristics at Community Level

**Figure 6:**
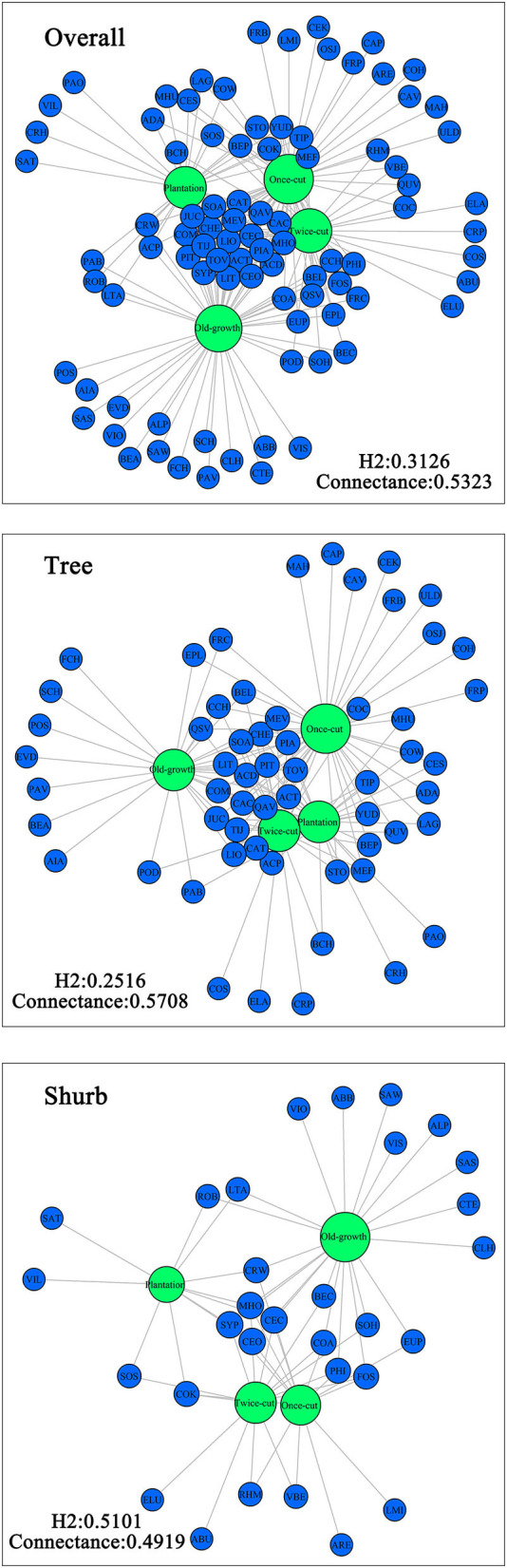
Network analysis of species composition among the four communities. The size of the dot indicates the abundance of species. Green circles indicate communities, and blue circles indicate species.

The Venn diagram showed significant differences in species composition among the different disturbance regimes of the forests. Four, 5, 11, and 15 species of woody plants were found in only one community in plantation forests, twice-cut forests, once-cut forests, and old-growth forests, respectively. The four communities shared 20 species. A total of 60.67% (54/89) of species occurred in two or more communities (Figure 5A). In the network analysis, the specialization index was 0.3126. The connectance index showed that the association between woody plants and different disturbance regimes forests was 53.23% (Figure 6).

### Species Specialization Characteristics at Species Level

The indicator species analysis showed that the indicator species of the four communities were different. The plantation forests were mainly composed of *Cerasus serrulata, Malus hupehensis*, and *Larix gmelinii*. The twice-cut forests were mainly composed of *Cornus kousa* subsp. *Chinensis, Rhododendron micranthum*, and *Corylus heterophylla*. The once-cut forests were mainly composed of *Betula platyphylla, Symplocos paniculata*, and *Quercus serrata* var. *brevipetiolata*. The old-growth forests were mainly composed of *Fraxinus chinensis, Ailanthus altissima*, and *Alangium platanifolium* (Table 2).

**Table 2:** Indicator species analysis in the four communities (*p* ≤ 0.05 level of significance).

**Species**	**Life form**	**Community**	***p*-value**
*Acer davidii* subsp. *grosseri*	Tree	A	0.05
*Malus hupehensis*	Tree	A	0.01
*Larix gmelinii*	Tree	A	0.01
*Cerasus serrulata*	Tree	A	0.01
*Juglans cathayensis*	Tree	A	0.01
*Styrax obassis*	Tree	A	0.01
*Cornus kousa* subsp. *chinensis*	Shurb	B	0.01
*Rhododendron micranthum*	Shurb	B	0.01
*Corylus heterophylla*	Tree	B	0.01
*Betula platyphylla*	Tree	C	0.01
*Symplocos paniculata*	Shurb	C	0.01
*Quercus serrata* var. *brevipetiolata*	Tree	C	0.01
*Carpinus polyneura*	Tree	C	0.04
*Carpinus turczaninowii*	Tree	C	0.01
*Tilia japonica*	Tree	C	0.01
*Pinus armandii Franch*	Tree	C	0.01
*Forsythia suspensa*	Shurb	C	0.01
*Toxicodendron vernicifluum*	Tree	C	0.01
*Carpinus cordata*	Tree	C	0.05
*Acer davidii*	Tree	C	0.01
*Lindera obtusiloba Blume*	Tree	C	0.01
*Tilia paucicostata*	Tree	C	0.01
*Sorbus alnifolia*	Tree	C	0.01
*Pinus tabuliformis*	Tree	C	0.01
*Fraxinus chinensis*	Tree	D	0.01
*Ailanthus altissima*	Tree	D	0.01
*Alangium platanifolium*	Shurb	D	0.01
*Malus honanensis Rehder*	Shurb	D	0.01
*Sorbus hupehensis*	Shurb	D	0.01
*Cotoneaster acutifolius*	Shurb	D	0.05
*Sambucus williamsii*	Shurb	D	0.01
*Abelia biflora*	Shurb	D	0.01
*Litsea tsinlingensis*	Tree	D	0.01
*Quercus aliena* var. *acutiserrata*	Tree	D	0.01
*Populus davidiana*	Tree	D	0.01
*Salix shihtsuanensis*	Shurb	D	0.01
*Euonymus phellomanus*	Shurb	D	0.01
*Acer pictum* subsp. *mono*	Tree	D	0.01
*Salix chaenomeloides Kimura*	Tree	D	0.01

*A, B, C, and D represent the plantation forest, twice-cut forest, once-cut forest, and old-growth forest, respectively*.

Based on the torus-translation test, 66.67% (38/57) species showed significant correlations (*P* < 0.05); among which 26 were positively correlated and 12 were negatively correlated (Table 3). In the 38 significant correlations, 31 species were associated with different communities. Seven species showed significant positive or negative correlations with different communities simultaneously. Twenty-six species showed no significant correlation with any habitat.

**Table 3:** Significant associations of woody plant species with the four communities (*P* ≤ 0.05 level of significance for torus-translation test).

**Species**	**Life form**	**Community**	**Species**	**Life form**	**Community**
*Betula platyphylla*	Tree	B(+) D(–)	*Celastrus orbiculatus*	Shurb	NA
*Fraxinus chinensis*	Tree	D(+)	*Meliosma veitchiorum*	Tree	D(–)
*Symplocos paniculata*	Shurb	B(+) D(–)	*Toxicodendron vernicifluum*	Tree	A(+) D(–)
*Ailanthus altissima*	Tree	D(+)	*Carpinus cordata*	Tree	D(–)
*Cornus controversa*	Tree	NA	*Litsea tsinlingensis*	Tree	D(+)
*Quercus serrata* var. *brevipetiolata*	Tree	NA	*Berberis circumserrata*	Shurb	D(+)
*Carpinus turczaninowii*	Tree	C(+) D(–)	*Acer davidii*	Tree	NA
*Acer davidii* subsp. *grosseri*	Tree	NA	*Quercus aliena* var. *acutiserrata*	Tree	C(–)
*Alangium platanifolium*	Shurb	D(+)	*Lindera obtusiloba*	Tree	NA
*Malus honanensis*	Shurb	C(–) D(+)	*Philadelphus incanus*	Shurb	D(+)
*Malus hupehensis*	Tree	NA	*Populus davidiana*	Tree	D(+)
*Sorbus hupehensis*	Shurb	D(+)	*Cerasus serrulata*	Tree	NA
*Lonicera tatarinowii*	Shurb	NA	*Tilia paucicostata*	Tree	C(+)
*Tilia japonica*	Tree	NA	*Salix shihtsuanensis*	Shurb	NA
*Pinus armandii*	Tree	C(+)	*Euonymus phellomanus*	Shurb	D(+)
*Corylus chinensis*	Tree	NA	*Quercus variabilis*	Tree	NA
*Crataegus wilsonii*	Shurb	NA	*Sorbus alnifolia*	Tree	NA
*Viburnum betulifolium*	Shurb	B(+)	*Cornus kousa* subsp. *chinensis*	Shurb	B(+) D(–)
*Cotoneaster acutifolius*	Shurb	D(+)	*Cerasus clarofolia*	Shurb	B(–)
*Viburnum opulus* var. *sargentii*	Shurb	NA	*Acer pictum* subsp. *mono*	Tree	B(–) D(+)
*Betula chinensis*	Tree	NA	*Salix chaenomeloides*	Tree	D(+)
*Sambucus williamsii*	Shurb	D(+)	*Juglans cathayensis*	Tree	NA
*Cornus macrophylla*	Tree	NA	*Pinus tabuliformis*	Tree	B(–)
*Forsythia suspensa*	Shurb	C(+)	*Yulania denudata*	Tree	NA
*Betula luminifera*	Tree	NA	*Styrax obassis*	Tree	NA
*Euptelea pleiosperma*	Tree	NA	*Acer truncatum*	Tree	NA
*Abelia biflora*	Shurb	D(+)	*Rhododendron micranthum*	Shurb	NA
*Larix gmelinii*	Tree	NA	*Corylus heterophylla*	Tree	B(+)
*Cornus walteri*	Tree	A(+)			

*A, B, C, and D represent the plantation forest, twice-cut forest, once-cut forest, and old-growth forest, respectively. NA represents no significant correlation. (+) indicates positive correlation, and (–) indicates negative correlation*.

### Differences in Species Specialization Characteristics Between Trees and Shrubs

The results of Kruskal–Wallis test showed significant differences in the species abundance and richness of trees and shrubs in the four communities (Figures 4C–F). The betadisper analysis followed by ANOVA test showed significant differences in the distribution of trees and shrubs in the four communities (Figures 5E,F). A total of 63.79% (37/58) of tree species were recorded in two or more communities (Figure 5B). A total of 54.84% (17/31) of shrub species were recorded in two or more communities (Figure 5C).

In the network analysis, for trees, the specialization index was 0.2516 and the connectance index was 0.5708. For shrubs, the specialization index was 0.5101 and the connectance index was 0.4919 (Figure 6). According to the torus-translation test, a total of 45.95% (17/37) species of tree species were associated with different communities. Fourteen species were positively correlated with communities, and 8 species were negatively correlated with communities (Table 3). A total of 70% (14/20) species of shrubs were associated with different communities. Twelve shrubs were positively correlated with communities, and four shrubs were negatively correlated with communities (Table 3).

## Discussion

In the network analysis, the specialization index of woody plants in the four forests of different disturbance regimes was 31.26%, which is higher than the previously reported plant–fungus network (0.265; Toju et al., 2014) and lower than the plant–seed diffusion network (0.354; Dicks et al., 2002). The findings suggest that ecological specialization plays an important role in the distribution of woody plant species. The characteristic network structure of plant communities may be determined by the biological relationships of species and environment (e.g., topography and soil) and interspecific relationship. Topography is an important environmental factor that reflects the soil environment, humidity, and temperature to some extent (Wangda and Ohsawa, 2006; Lan et al., 2011; Lei, 2019). However, in the present study, topographic factors played a small role in determining the distribution of species among forests of different disturbance regimes (Supplementary Figure 1). The interspecific relationship in the forests mainly had a negative correlation (Supplementary Figures 2–5). Therefore, interspecific relationship may be partly responsible for the observed moderate modularity in plant–community networks. Hence, the distribution of woody plants among forests of different disturbance regimes is not random but is specialized.

The indicator species analysis showed variations in the assemblage characteristics of woody plants in different disturbance regimes of forests (Table 2). The torus-translation test showed that half of the species had significant correlations with different communities (38/57, 66.67%). Therefore, different woody plants show different community preferences in forest of different disturbance regimes. The torus translation showed that 38.60% (22/57) of the species preferred to be distributed in the old-growth forests, while only 3.51% (2/57) of the species were distributed in the plantation forests. This finding is due to the fact that the plantation forest is far more human disturbed than the undisturbed forest, which is undisturbed for a long time, so more habitats will be formed (Gao et al., 2017). Twelve species showed a negative association with the old-growth forests (Table 3), where these species are eliminated from the communities because of long-term environmental filtering (Yan and Bi, 2009). For example, *Betula platyphylla* is a fast-growing and dominant species in the early stages of community succession. In the late stage of succession, diseases and insect pests often occur due to lack of light and other reasons, and this species is eventually eliminated from the community (Zhu, 1994; Dong et al., 2005; Guo et al., 2007; Yang et al., 2007). Our study demonstrates the importance of forest partitioning with different disturbance regimes in maintaining local diversity in a woody plant community.

Consistent with our hypothesis, shrubs showed higher specialization than trees in forests of different disturbance regimes. The specialization index of shrubs (51.01%) is higher than that of trees (25.16%). In addition, more shrub species (70.00%) had specific preferences than tree species (45.95%) with respect to forests in different disturbance regimes. In addition to topography, soil physical and chemical properties, interspecific relationships, and other environments, shrub species are more affected by forest canopy structure than tree species (Hu et al., 2019). Great differences in canopy structure were found among different disturbance regimes of forests, resulting in differences in light environment, soil physical and chemical properties, and litter under the forest (Song et al., 2015; Gao et al., 2017; Han et al., 2017; Gallé et al., 2018). Diverse habitats under the forest canopy provide suitable environments for the growth of different shrub species (Yuan et al., 2012; Jia et al., 2019). Therefore, more shrub species exhibited distinct community preferences than tree species in forests of different disturbance regimes.

## Conclusions and Implications

Our study finds that the distribution of woody plants among forests with different disturbance regimes is not random but specialized. Different woody plants show different community preferences in different disturbance regimes of forests. Shrubs have higher specialization than trees in forests of different disturbance regimes.

In terms of forest sustainable development, woody plant species should not be randomly planted in the forest, but species that prefer to be distributed in forests of different disturbance regimes should be considered. According to our results, for example, *Sorbus hupehensis* prefers to be distributed in the old-growth forest, *Viburnum betulifolium* prefers to be distributed in the twice-cut forest, and *Malus honanensis* does not like to be distributed in the once-cut forest (Table 3). In addition, more attention has been paid to the distribution preference of shrub species to forests in different disturbance regimes than to tree species.

## Data Availability Statement

The original contributions presented in the study are included in the article/Supplementary Materials, further inquiries can be directed to the corresponding author/s.

## Author Contributions

YC and ZY conceived the ideas. JX developed methodology and led the writing of the manuscript. JX, YS, ZL, PZ, YY, WL, ZY, and YC conducted fieldwork. All authors contributed to the article and approved the submitted version.

## Conflict of Interest

The authors declare that the research was conducted in the absence of any commercial or financial relationships that could be construed as a potential conflict of interest.

## References

[B1] Backer A. D. Hoey G. V. Coates D. Vanaverbeke J. Hostens K. (2014). Similar diversity-disturbance responses to different physical impacts: three cases of small-scale biodiversity increase in the Belgian part of the North Sea. Mar. Pollut. Bull. 84, 251–262. 10.1016/j.marpolbul.2014.05.006 24889315

[B2] Barlow J. Gardner T. A. Louzada J. Peres C. A. (2010). Measuring the conservation value of tropical primary forests: the effect of occasional species on estimates of biodiversity uniqueness. PLoS One 5:e9609. 10.1371/journal.pone.0009609 20231897 PMC2834753

[B3] Bevill R. L. Louda S. M. (1999). Comparisons of related rare and common species in the study of plant rarity. Conserv. Biol. 13, 493–498. 10.1046/j.1523-1739.1999.97369.x

[B4] Bi H. T. Wang B. Y. Yang Z. H. Feng J. Zhang J. H. Gao M. X. (2014). Study on the dynamic change of the tree layer biomass of the community *Quercus aliena* var.acutiserrata-Pinus armandii of Baiyunshan Mountain in Henan Province. J. Henan Agric. Univ. 48, 736–740. 10.16445/j.cnki.1000-2340.2014.06.019

[B5] Blüthgen N. Menzel F. Hovestadt T. Fiala B. Blüthgen N. (2007). Specialization, constraints, and conflicting interests in mutualistic networks. Curr. Biol. 17, 341–346. 10.1016/j.cub.2006.12.039 17275300

[B6] Burrascano S. Keeton W. S. Sabatini F. M. Blasi C. (2013). Commonality and variability in the structural attributes of moist temperate old-growth forests: a global review. For. Ecol. Manage. 291, 458–479. 10.1016/j.foreco.2012.11.020

[B7] Chazdon R. L. (2003). Tropical forest recovery: legacies of human impact and natural disturbances. Perspect. Plant Ecol. Evol. Syst. 6, 51–71. 10.1078/1433-8319-00042

[B8] Chen Y. (2015). The Maintaining Mechanism Study of Vascular Plants Diversity in Xiaoqinling Nature Reserve. Henan Agricultural University.

[B9] Chen Y. Shao Y. Xi J. Yuan Z. Ye Y. Wang T. (2020). Community preferences of woody plant species in a heterogeneous temperate forest, China. Front. Ecol. Evol. 8:165. 10.3389/fevo.2020.00165

[B10] Chen Y. Yuan Z. L. Ren S. Y. Wei B. L. Jia H. R. Ye Y. Z. (2014). Correlation analysis of soil and species of different life forms in Baotianman Nature Reserve (in Chinese). Chin. Sci. Bull. 59, 2367–2376. 10.1360/N972014-00323

[B11] Comita L. S. Condit R. Hubbell S. P. (2007). Developmental changes in habitat associations of tropical trees. J. Ecol. 95, 482–492. 10.1111/j.1365-2745.2007.01229.x24812111

[B12] Condit R. (1995). Research in large, long-term tropical forest plots. Trends Ecol. Evol. 10, 18–22. 10.1016/S0169-5347(00)88955-7 21236939

[B13] De Cáceres M. (2013). How to Use the Indicspecies Package (ver. 1.7. 1). Centre Tecnològic Forestal de Catalunya, Catalonia.

[B14] Debski I. Burslem D. F. R. P. Palmiotto P. A. Lafrankie J. V. Lee H. S. Manokaran N. (2002). Habitat preferences of Aporosa in two Malaysian forests:implications for abundance and coexistence. Ecology 83, 2005–2018. 10.1890/0012-9658(2002)083[2005:HPOAIT]2.0.CO;2

[B15] Devictor V. Clavel J. Julliard R. Lavergne S. Mouillot D. Thuiller W.. (2010). Defining and measuring ecological specialization. J. Appl. Ecol. 47, 15–25. 10.1111/j.1365-2664.2009.01744.x

[B16] DeWalt S. J. Ickes K. Nilus R. Harms K. E. Burslem D. F. R. P. (2006). Liana habitat associations and community structure in a Bornean lowland tropical forest. Plant Ecol. 186, 203–216. 10.1007/s11258-006-9123-6

[B17] Dicks L. V. Corbet S. A. Pywell R. F. (2002). Compartmentalization in plant-insect flower visitor webs. J. Anim. Ecol. 71, 32–43. 10.1046/j.0021-8790.2001.00572.x

[B18] Dong J. H. Xue Q. H. Zhang J. C. Hao F. (2005). Soil fertility characteristic and mixed effect of plantation forests on loess plateau. J. Northwest For. Univ. 20, 31–35.

[B19] Dormann C. F. Fründ J. Blüthgen N. Gruber B. (2009). Indices, graphs and null models: analyzing bipartite ecological networks. Open Ecol. J. 2, 7–24. 10.2174/1874213000902010007

[B20] Fan D. X. Yu X. X. (2016). Spatial point pattern analysis of *Quercus variabilis* and *Pinus tabulaeformis* populations in a mountainous area of Beijing. Acta Ecol. Sin. 36, 318–325. 10.5846/stxb201402060215

[B21] Fang Z. Y. Li L. Y. Maola A. K. E. Zhou L. Lu B. (2019). Effects of human disturbance on plant diversity of wild fruit forests in Western Tianshan Mountain. Bull. Soil Water Conserv. 39, 267–374.

[B22] Gallé Á. Czékus Z. Bela K. Horváth E. Ördög A. Csiszár J.. (2018). Plant glutathione transferases and light. Front. Plant Sci. 9:1944. 10.3389/fpls.2018.01944 30687349 PMC6333738

[B23] Gao C. Shi N. N. Chen L. Ji N. N. Wu B. W. Wang Y. L.. (2017). Relationships between soil fungal and woody plant assemblages differ between ridge and valley habitats in a subtropical mountain forest. New Phytol. 213, 1874–1885. 10.1111/nph.14287 28164340

[B24] Gunatilleke C. V. S. Gunatilleke I. A. U. N. Esufali S. Harms K. E. Ashton P. M. S. Burslem D. F. R. P.. (2006). Species-habitat associations in a Sri Lankan dipterocarp forest. J. Trop. Ecol. 22, 371–384. 10.1017/S0266467406003282

[B25] Guo Q. Q. Zhang W. H. He J. F. Wang Z. H. (2007). Structural characteristics of different betula platyphylla communities in Huanglong Mountain. Acta Bot. Boreali-Occidentalia Sin. 1, 132–138.

[B26] Han B. C. Umaña M. N. Mi X. C. Liu X. J. Chen L. Wang Y. Q.. (2017). The role of transcriptomes linked with responses to light environment on seedling mortality in a subtropical forest, China. J. Ecol. 105, 592–601. 10.1111/1365-2745.12760

[B27] Hao J. F. Wang D. Y. Li Y. Yao X. L. Zhang Y. B. Zhan M. C.. (2014). Effects of human disturbance on species diversity of Phoebe zhennan communitis in Jinfengshan Moutain in western Sichuan. Acta Ecol. Sin. 34, 6930–6942. 10.5846/stxb201401140103

[B28] Harms K. E. Condit R. Hubbell S. P. Foster R. B. (2001). Habitat associations of trees and shrubs in a 50-ha Neotropical forest plot. J. Ecol. 89, 947–959. 10.1111/j.1365-2745.2001.00615.x

[B29] Hu W. J. Pan L. Lei J. P. Tang W. P. Pang H. D. Cui H. X.. (2019). Effects of forest stand structure characteristics on shrub species diversity in *Pinus massoniana* forest in Three Gorges reservoir are. Ecol. Environ. Sci. 28, 1332–1340. 10.16258/j.cnki.1674-5906.2019.07.006

[B30] Jia H. R. Chen Y. Wang X. Y. Li P. K. Yuan Z. L. Ye Y. Z. (2019). The relationships among topographically-driven habitats, dominant species and vertical layers in temperate forest in China. Russ. J. Ecol. 50, 172–186. 10.1134/S1067413619020061

[B31] Lan G. Hu Y. Cao M. Zhu H. (2011). Topography related spatial distribution of dominant tree species in a tropical seasonal rain forest in China. For. Ecol. Manag. 262, 1507–1513. 10.1016/j.foreco.2011.06.052

[B32] Laurance W. F. Peres C. A. (2006). Emerging Threats to Tropical Forests. Chicago, IL: University of Chicago Press.

[B33] Laurance W. F. Sayer J. Cassman K. G. (2014). Agricultural expansion and its impacts on tropical nature. Trends Ecol. Evol. 29, 107–116. 10.1016/j.tree.2013.12.001 24388286

[B34] Lei S. Y. (2019). Vegetation Restoration With Soil Physical and Chemical Properties' Distribution Characteristics of Converted Grassland in Different Topographic Conditions. Journal of Northwest A&F University.

[B35] Lhotka J. M. Loewenstein E. F. (2008). Influence of canopy structure on the survival and growth of underplanted seedlings. New For. 35, 89–104. 10.1007/s11056-007-9063-6

[B36] Li L. H. Yang H. Z. Wang J. Qin Y. F. Fu Q. J. (2017). Species structure and diversity of plant community of Luhua valley in Baiyun Mountain of Henan Province. Hunan Agric. Sci. 10, 44–47. 10.16498/j.cnki.hnnykx.2017.010.013

[B37] Liu Z. M. Zhao X. Y. Liu X. M. (2002). Relationship between disturbance and vegetation. Acta Pratacult. Sin. 11, 1–9.

[B38] Lü G. Wang T. Li Y. X. Wei Z. P. Wang K. (2017). Herbaceous plant diversity and soil physicochemical properties on the regeneration slash of *Pinus sylvestris* var.mongolica. Acta Ecol. Sin. 37, 8294–8303. 10.5846/stxb201610162093

[B39] Lu T. Ma K. M. Ni H. W. Fu B. J. Zhang J. Y. Lu Q. (2008). Variation in species composition and diversity of wetland communities under different disturbance intensity in the Sanjiang Plain. Acta Ecol. Sin. 28, 1893–1900. 10.1016/S1872-2032(08)60040-2

[B40] Oksanen J. Kindt R. Legendre P. O'Hara B. Stevens M. H. H. Oksanen M. J.. (2007). Vegan: Community Ecology Package. Available online at: https://cran.r-project.org/web/packages/vegan/index.html (accessed September 1, 2019).

[B41] Parkke W. C. Det D. C. (2008). Influence of overstory density on ecophysiology of red oak (*Quercus rubra*) and sugar maple (*Acer saccharum*) seedlings in central Ontario shelter woods. Tree Physiol. 28, 797–804. 10.1093/treephys/28.5.797 18316311

[B42] Pierre L. (2007). Studying beta diversity: ecological variation partitioning by multiple regression and canonical analysis. Chin. J. Plant Ecol. 31, 976–981. 10.17521/cjpe.2007.0124

[B43] Poisot T. Bever J. D. Nemri A. Thrall P. H. Hochberg M. E. (2011). A conceptual framework for the evolution of ecological specialisation. Ecol. Lett. 14, 841–851. 10.1111/j.1461-0248.2011.01645.x 21699641 PMC3152695

[B44] Potts M. D. Ashton P. S. Kaufman L. S. Plotkin J. B. (2002). Habitat patterns in tropical rain forests: a comparison of 105 plots in northwest Borneo. Ecology 83, 2782–2797. 10.1890/0012-9658(2002)083[2782:HPITRF]2.0.CO;2

[B45] Singh S. P. (1998). Chronic disturbance, a principal cause of environmental degradation in developing countries. Environ. Conserv. 25, 1–2. 10.1017/S0376892998000010

[B46] Song P. Ren H. Jia Q. Guo J. Zhang N. Ma K. (2015). Effects of historical logging on soil microbial communities in a subtropical forest in southern China. Plant Soil, 397, 115–126. 10.1007/s11104-015-2553-y

[B47] Toju H. Guimaraes P. R. Olesen J. M. Thompson J. N. (2014). Assembly of complex plant-fungus networks. Nat. Commun. 5:5273. 10.1038/ncomms6273 25327887 PMC4218951

[B48] Wang X. Y. Cao R. F. Fan P. Z. Yuan Z. L. Ye Y. Z. (2018). Relationship between macrofungal diversity and environment in Baiyun Mountain forest park. J. Henan Agric. Univ. 52, 604–610. 10.16445/j.cnki.1000-2340.2018.04.019

[B49] Wangda P. Ohsawa M. (2006). Structure and regeneration dynamics of dominant tree species along altitudinal gradient in a dry valley slopes of the Bhutan Himalaya. For. Ecol. Manage. 230, 136–150. 10.1016/j.foreco.2006.04.027

[B50] Yamada T. Tomita A. Itoh A. Yamakura T. Ohkubo T. Kanzaki M.. (2006). Habitat associations of Sterculiaceae trees in a Bornean rain forest plot. J. Veg. Sci. 17, 559–566. 10.1658/1100-9233(2006)17[559:HAOSTI]2.0.CO;2

[B51] Yan M. Bi R. C. (2009). Vegetation classification and comparative analysis of species diversity of community at different succession stages in Huoshan Mountain of Shanxi Province. J. Plant Resour. Environ. 18, 56–62.

[B52] Yang H. Lou A. R. Gao Y. J. Song H. T. (2007). Liff history characteristics and spatial distribution of the betula platyphulla population in the Dongling Mountain region, Beijing, China. Chin. J. Plant Ecol. 31, 272–282. 10.17521/cjpe.2007.0031

[B53] Ye L. Q. (2000). The relationship between disturbance and biodiversity. J. Guizhou Univ. 17, 129–134. 10.3969/j.issn.1000-5269.2000.02.010

[B54] Yuan Z. Q. Gazol A. Wang X. G. Xing D. L. Lin F. Bai X. J.. (2012). What happens below the canopy? Direct and indirect influences of the dominant species on forest vertical layers. Oikos 121, 1145–1153. 10.1111/j.1600-0706.2011.19757.x

[B55] Zhang X. P. Shangguan Z. P. (2006). Effect of Human-induced disturbance on physical properties of soil in artificial *Pinus tabulaeformis* Carr. forests of the Loess Plateau. Acta Ecol. Sin. 26, 3685–3696. 10.1016/S1872-2032(06)60008-5

[B56] Zhu Z. C. (1994). Prelirnanary studies on theBetula platyphyllaforests in loess plateau of northern Shaanxi Province. J. Northwest Univ. 24, 455–459.

