# Supplementary material for: Distribution of Woody Plant Species Among Different Disturbance Regimes of Forests in a Temperate Deciduous Broad-Leaved Forest
Source: Front Plant Sci. 2021 Apr 6;12:618524. doi: 10.3389/fpls.2021.618524 (PMC8056040; doi:10.3389/fpls.2021.618524)
Supplement: Supplementary file 1 [file Data_Sheet_1.docx]

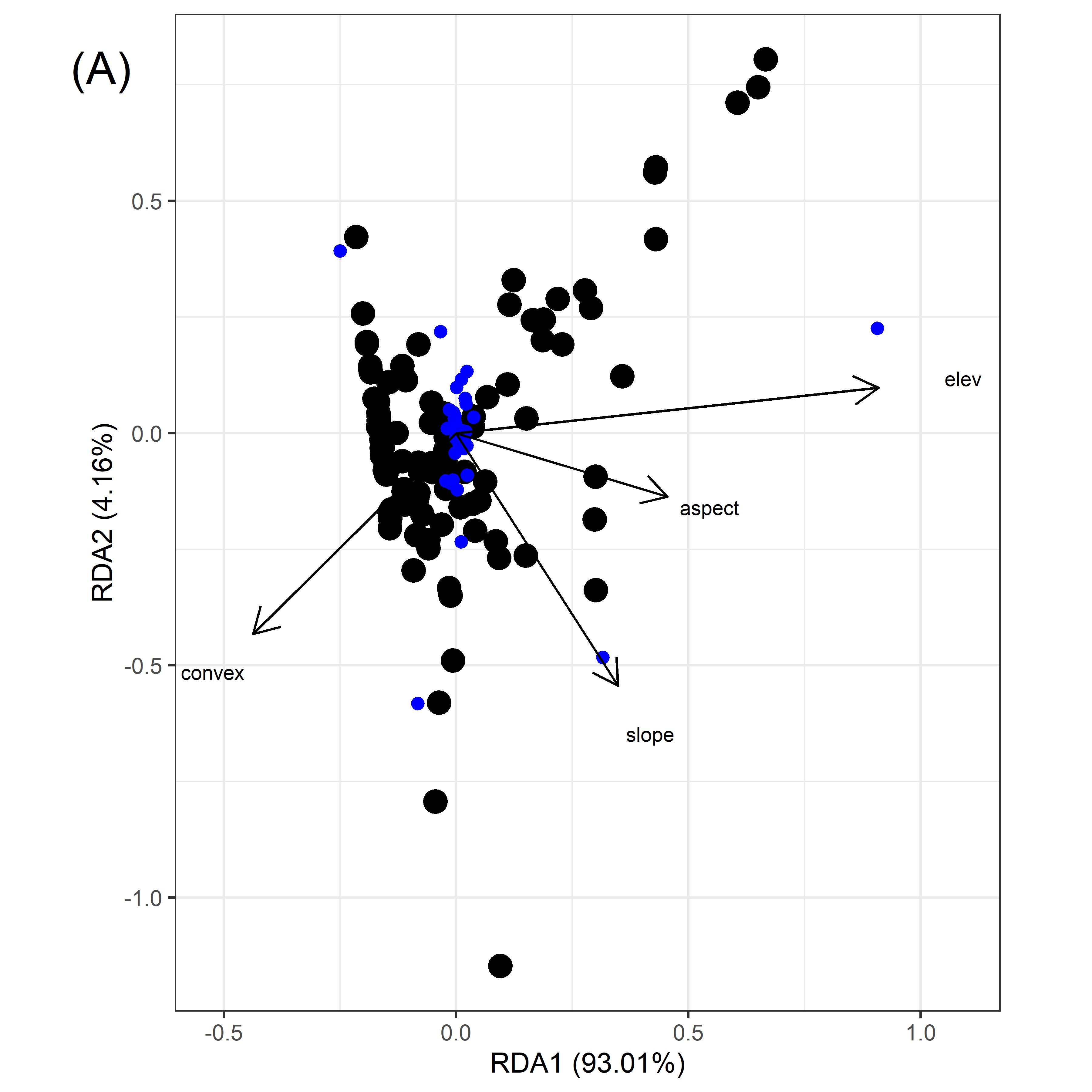

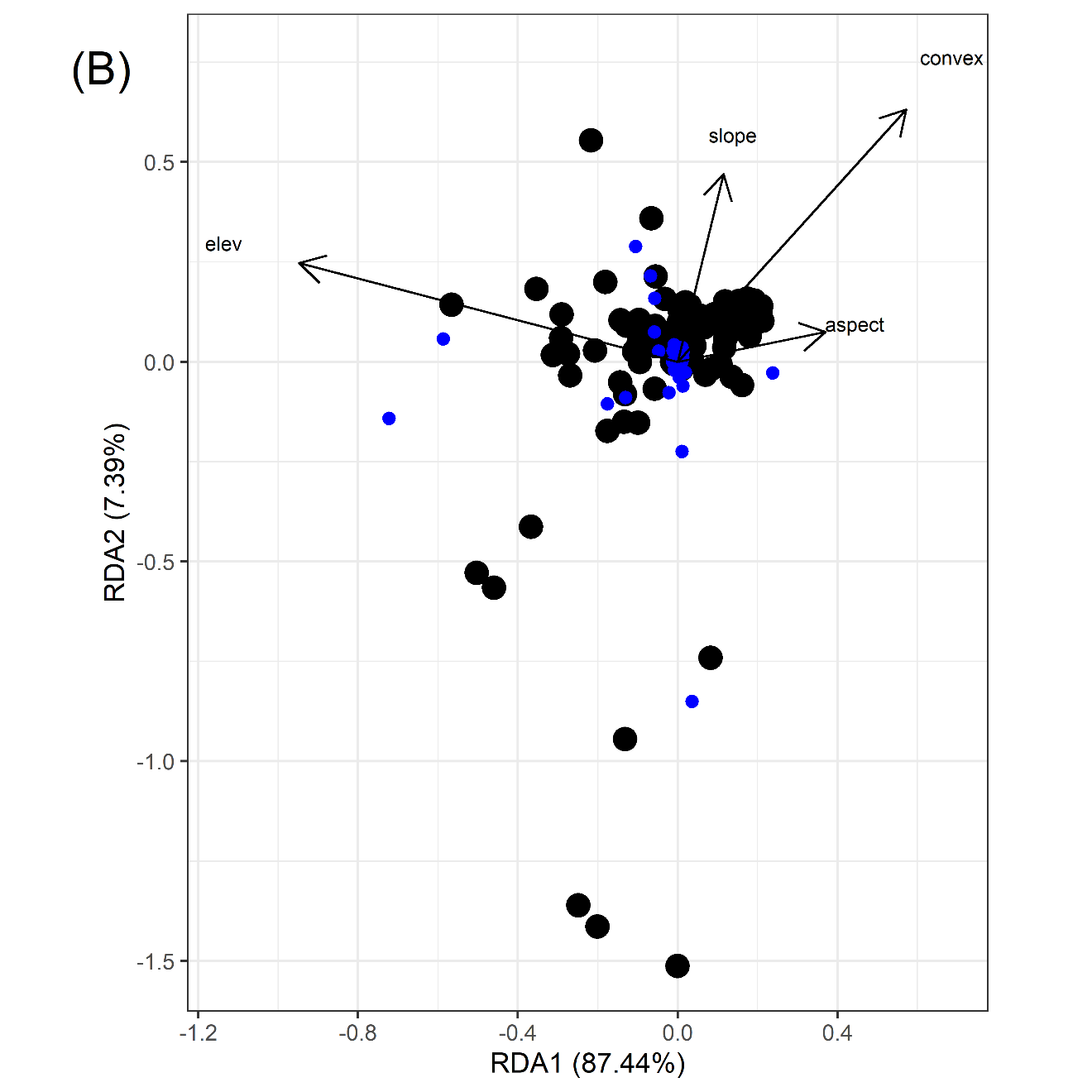


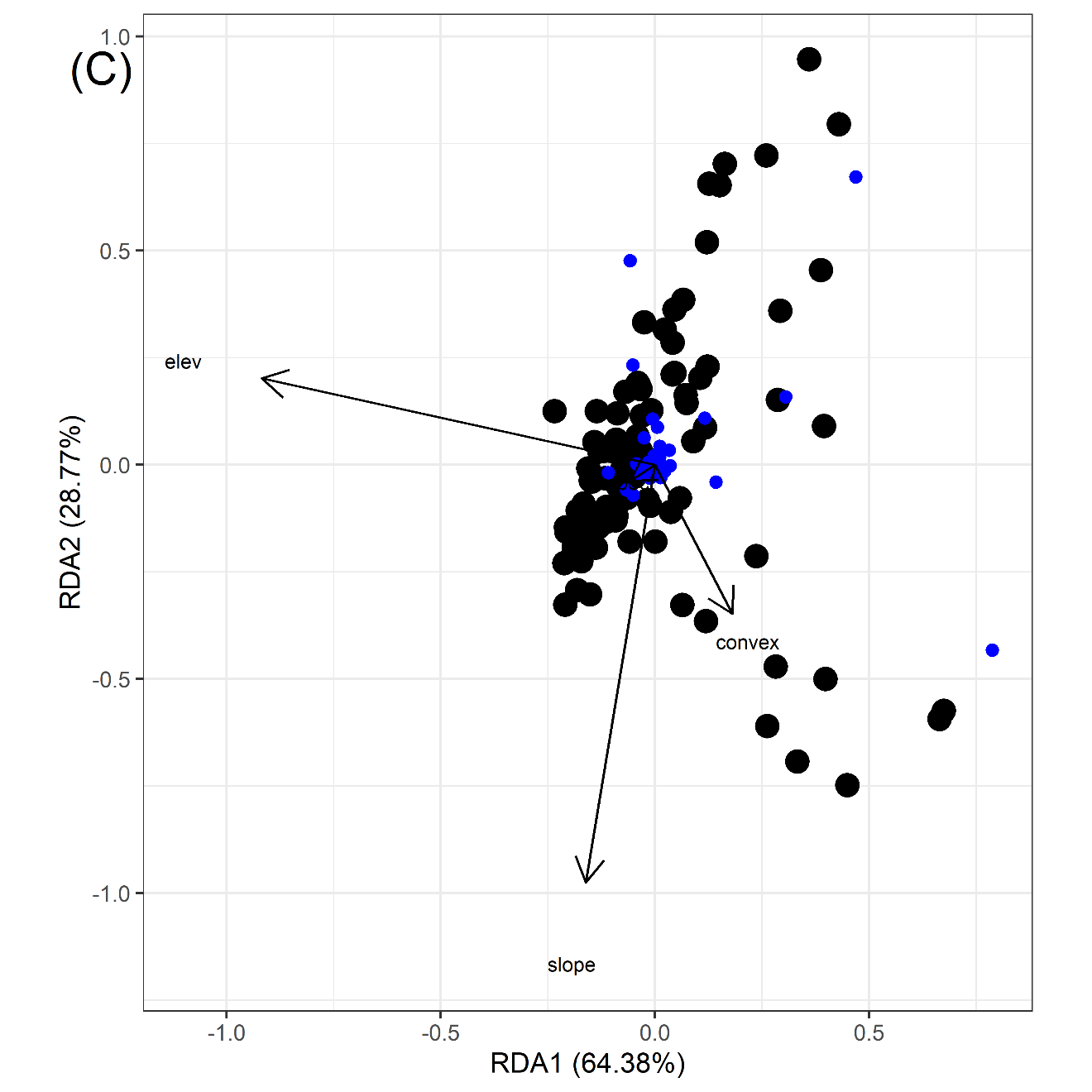

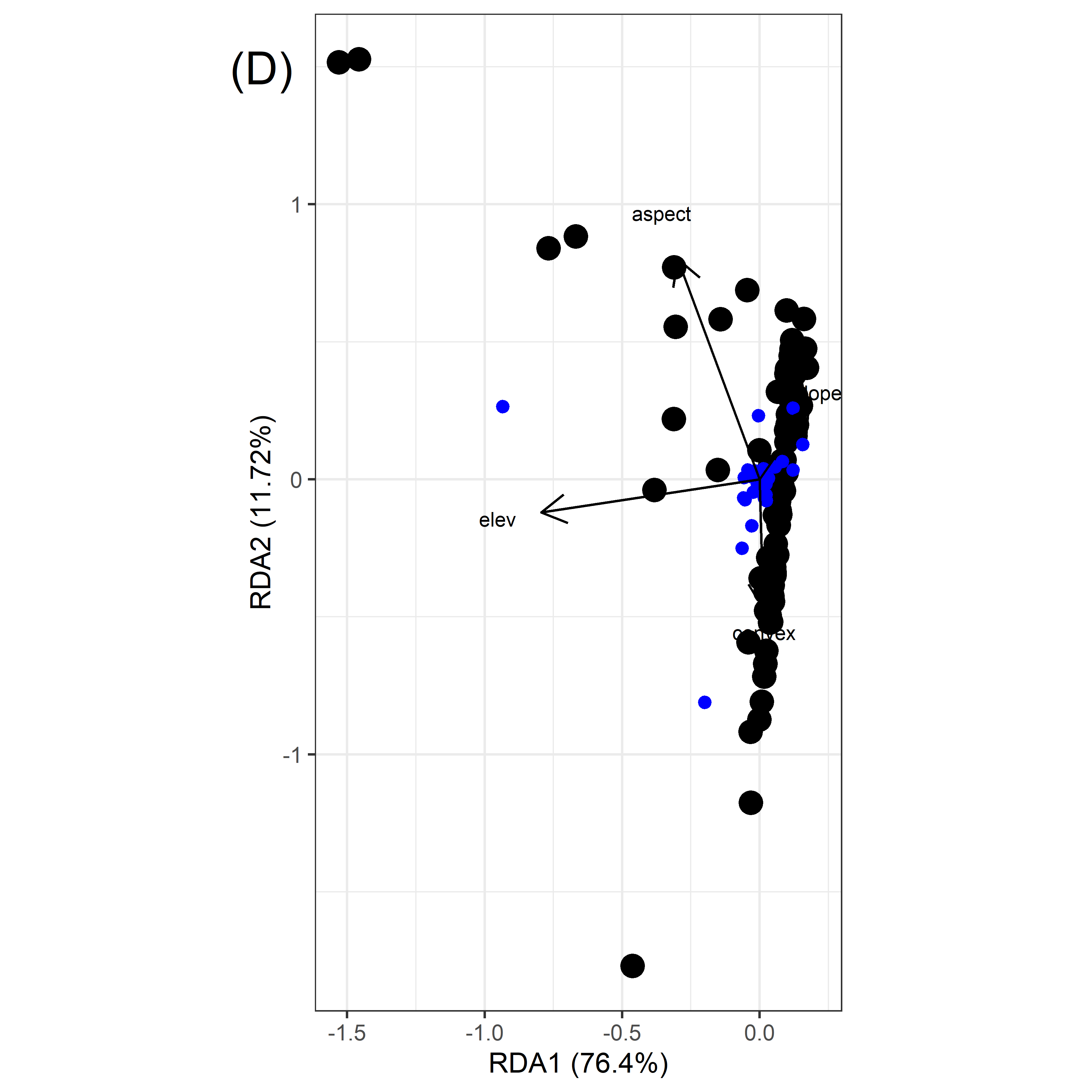


**Figure S1** RDA ordination diagram of four communities and topographic factors (Elevation (m), convex concave (°), slope (°), and aspect (°) ). A, B, C, and D represent the plantation forest, twice-cut forest, once-cut forest, and old growth forest, respectively.


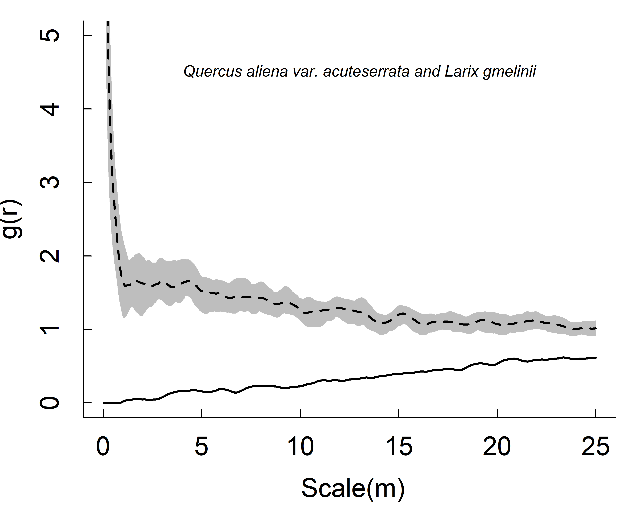

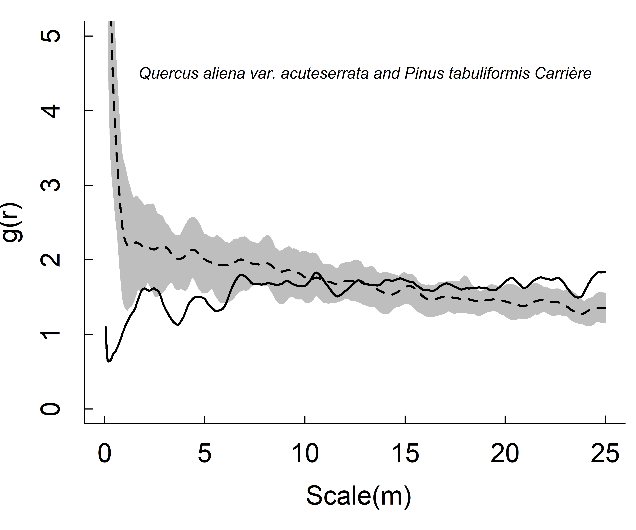


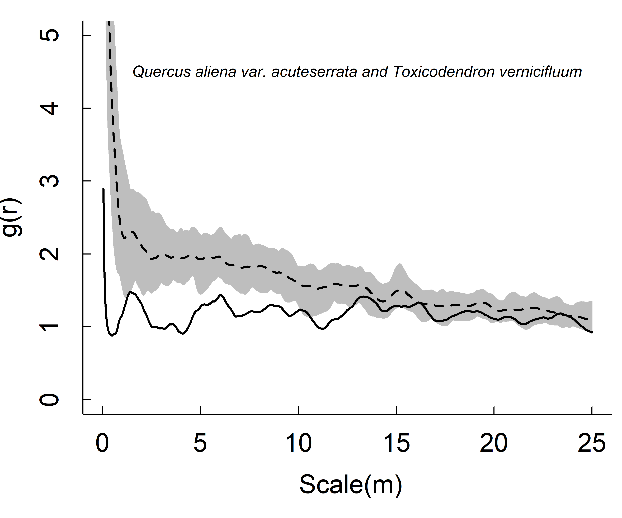

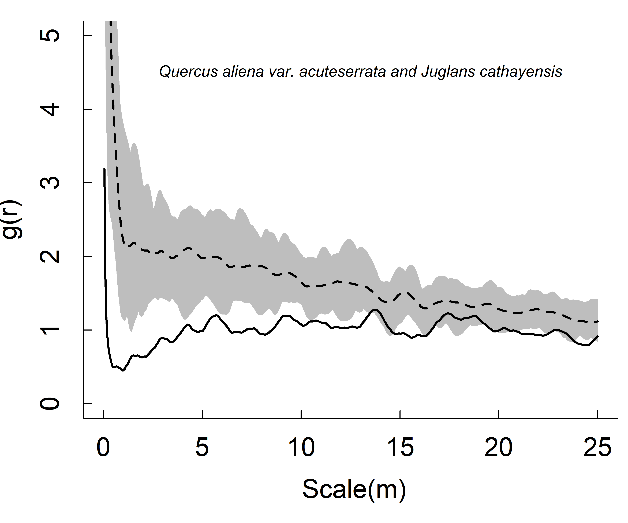


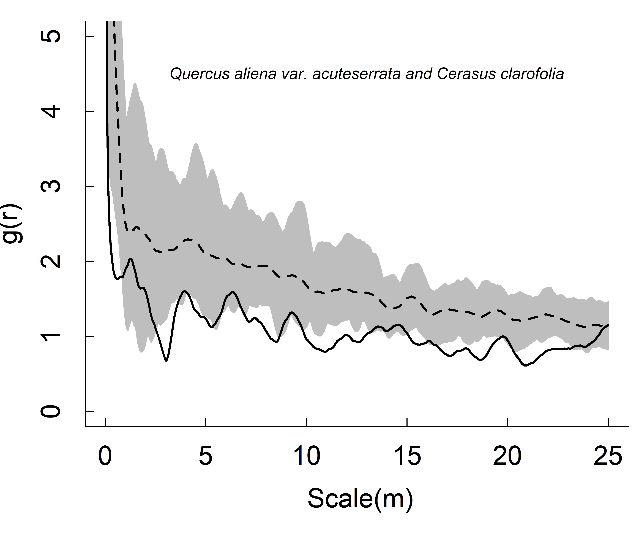

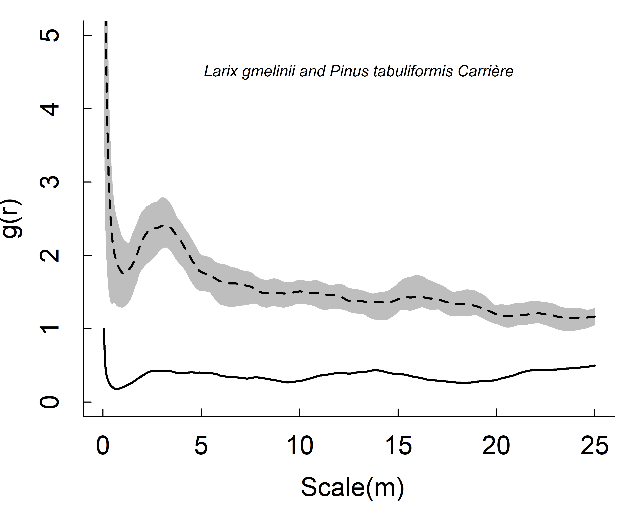


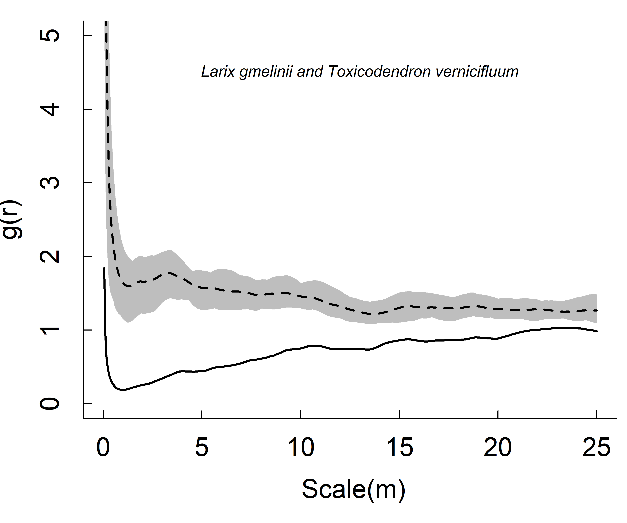

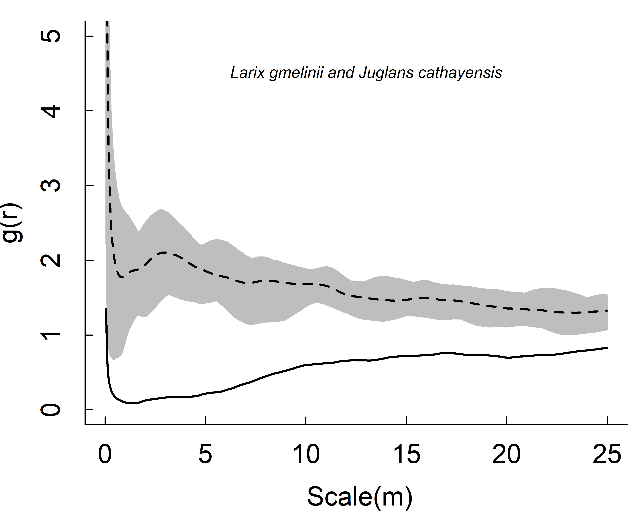


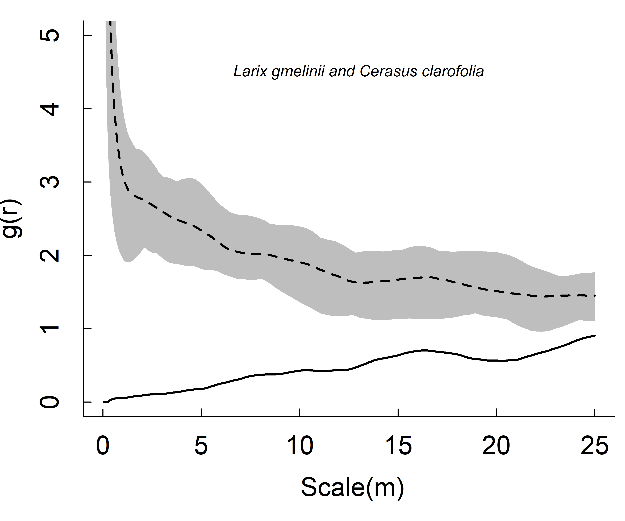

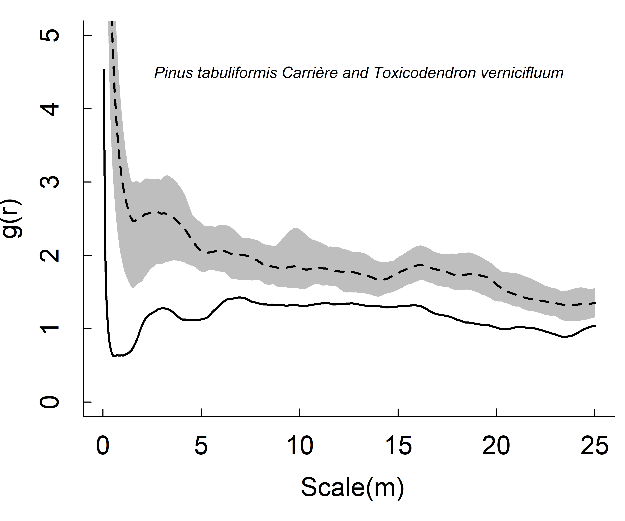


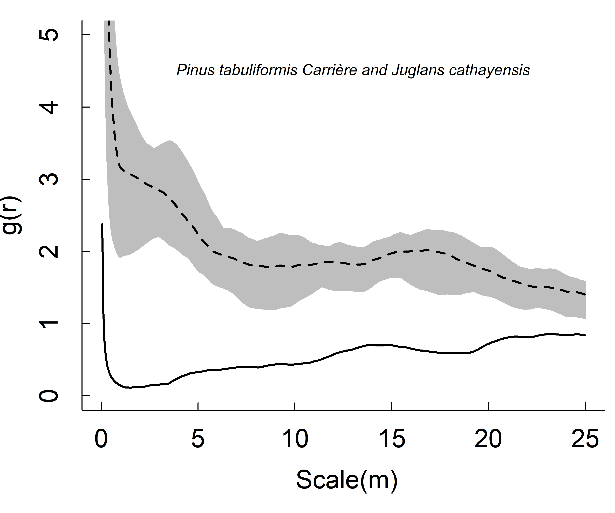

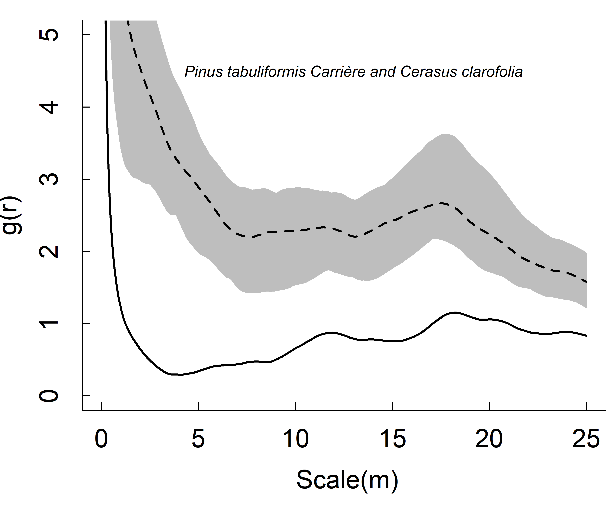


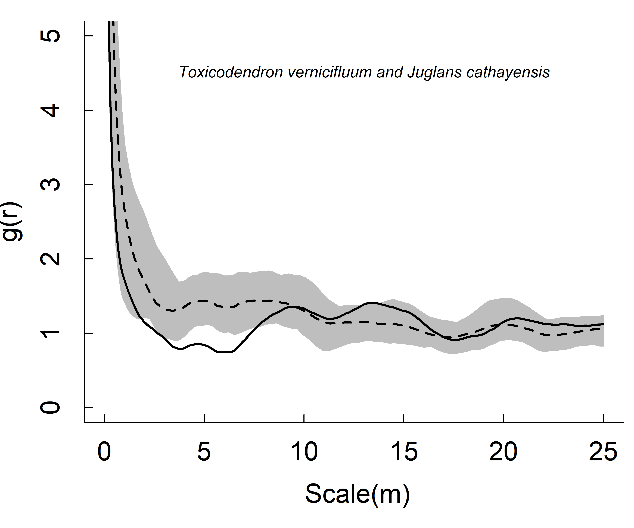

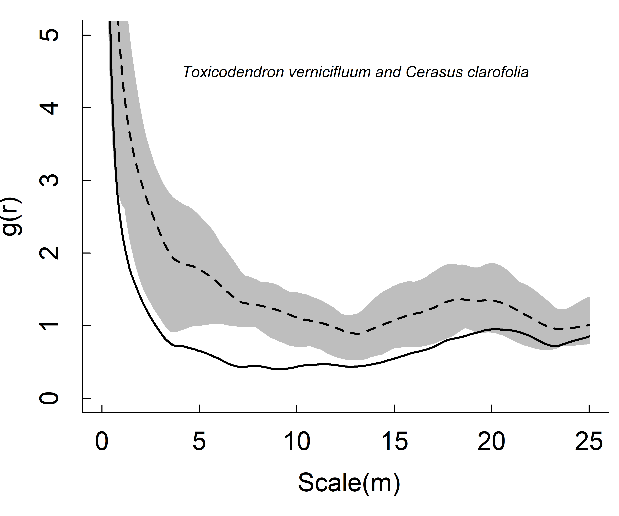


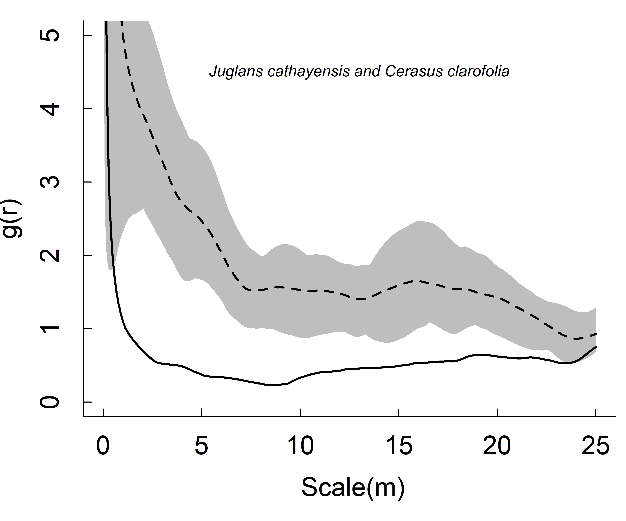


**Figure S2** Analysis of point pattern of six dominant species in the plantation forest (1 hm^2^ sample plot). The selected function was g(r) function. The solid line denotes the actual interspecific values of the two species, the dashed line denotes the theoretical values, and the gray interval denotes the confidence interval. When g (r) is within the confidence interval, the two species are independent of each other. When g (r) value is above the confidence interval, the two species are significantly positively correlated. When the g (r) value is below the confidence interval, the two species are significantly negatively correlated.


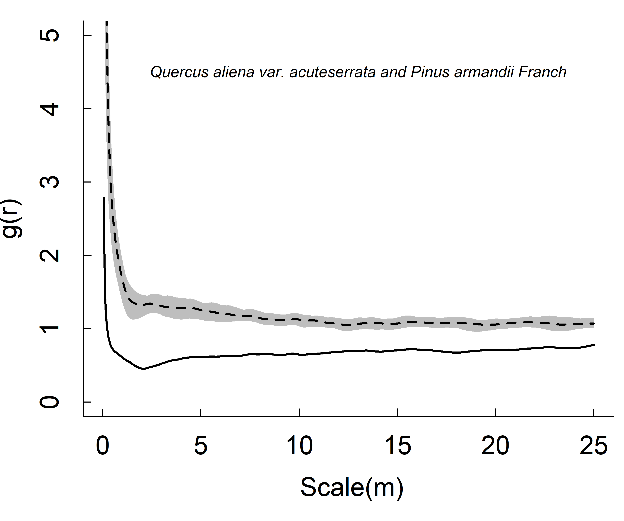

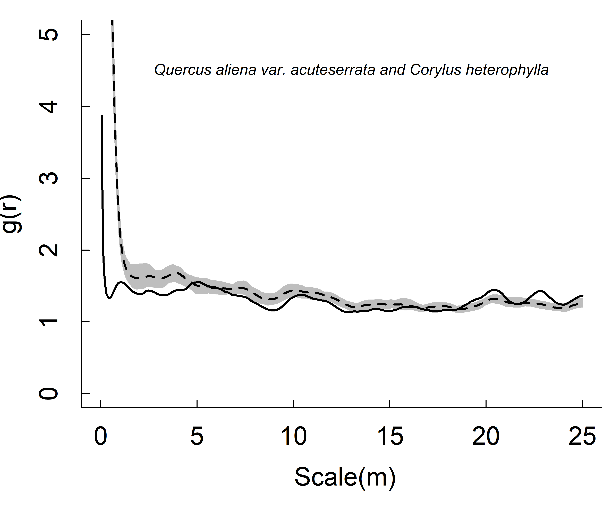


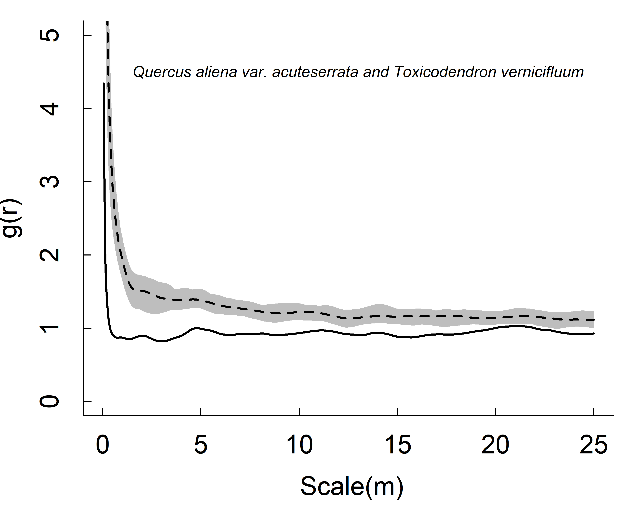

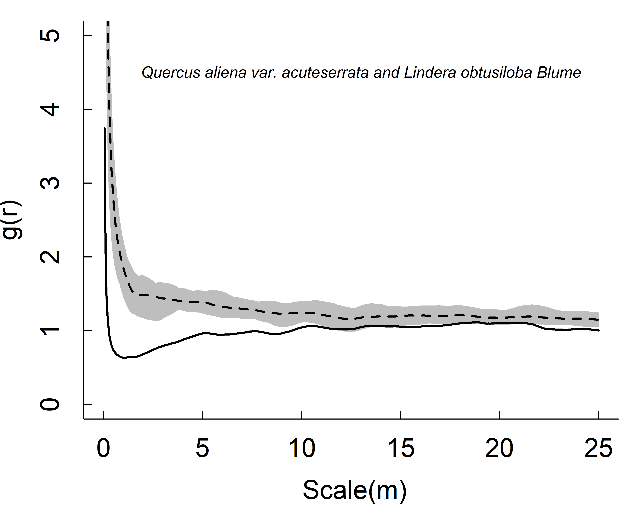


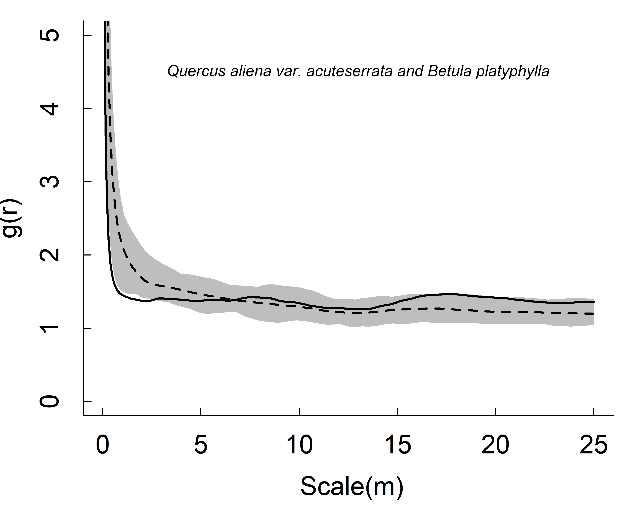

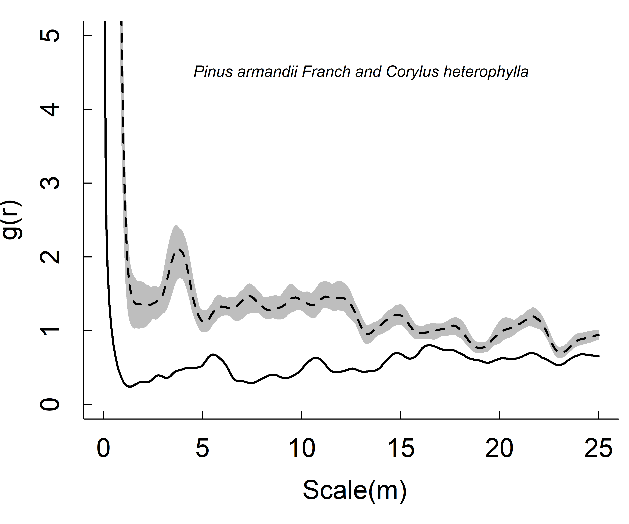


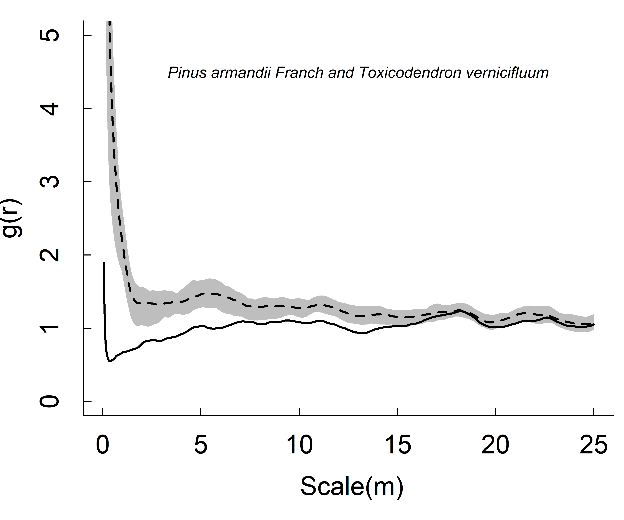

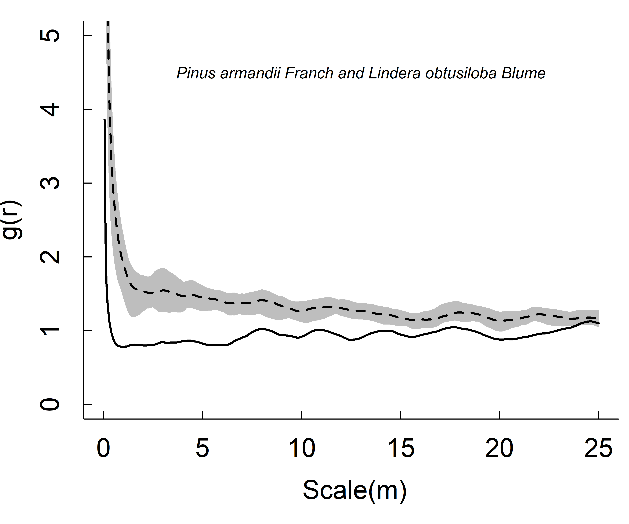


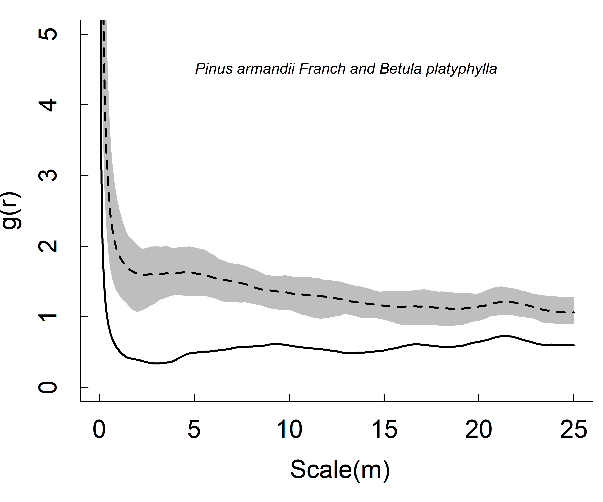

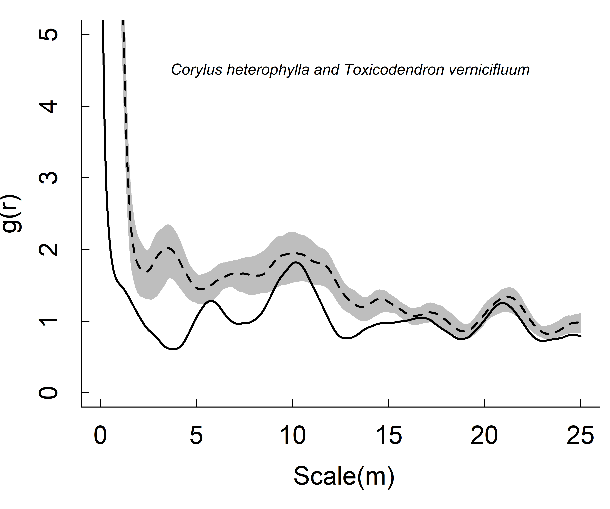


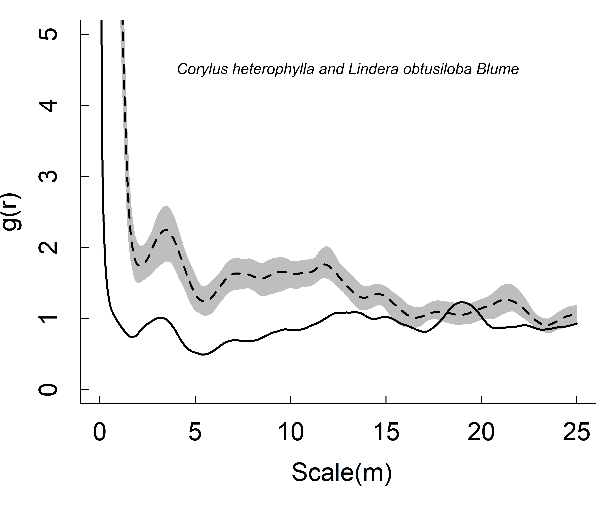

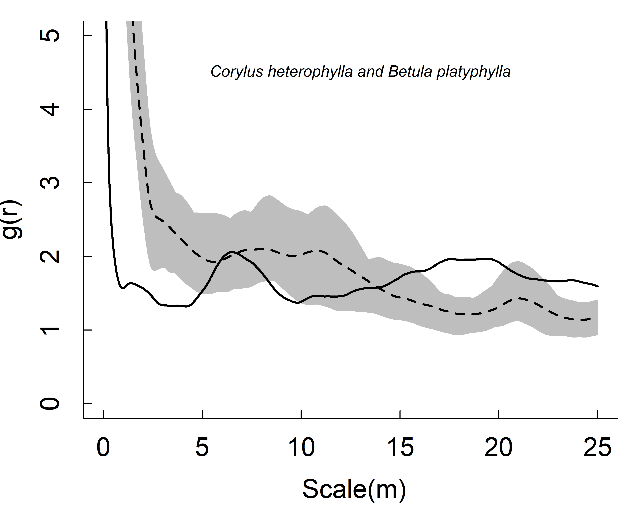


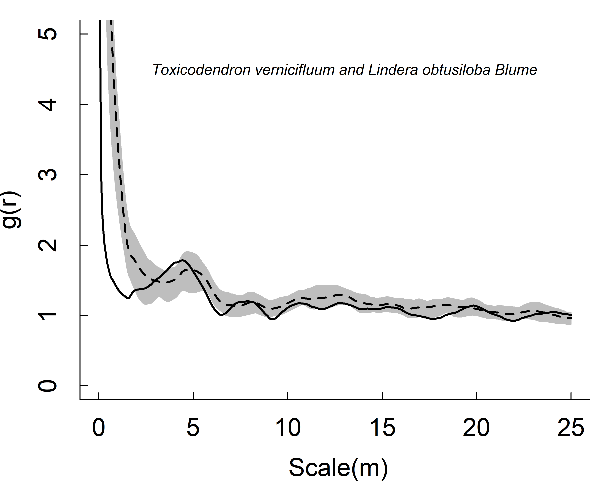

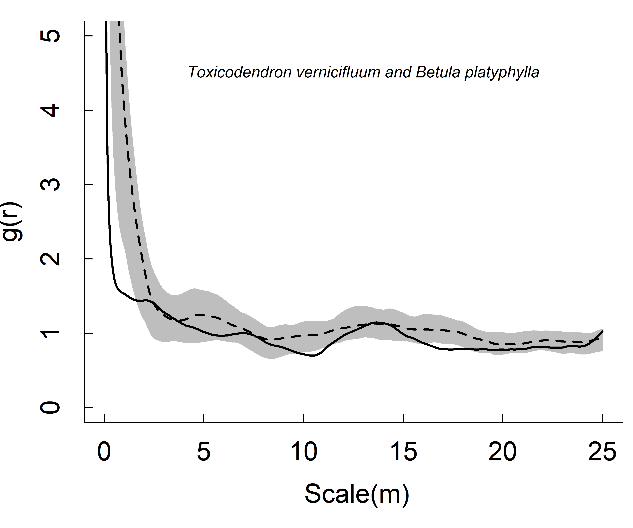


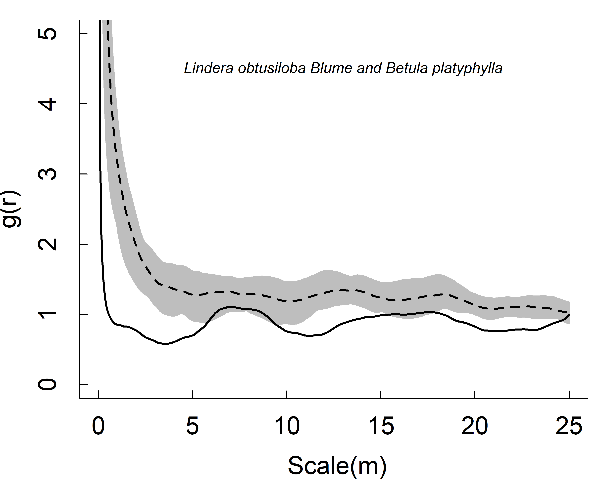


**Figure S3** Analysis of point pattern of 6 dominant species in twice-cut forest 1hm^2^ sample plot. The selected function was g(r) function. The solid line denotes the actual interspecific values of the two species, the dashed line denotes the theoretical values, and the gray interval denotes the confidence interval. When g (r) is within the confidence interval, the two species are independent of each other. When g (r) value is above the confidence interval, the two species are significantly positively correlated. When the g (r) value was below the confidence interval, the two species were significantly negatively correlated.


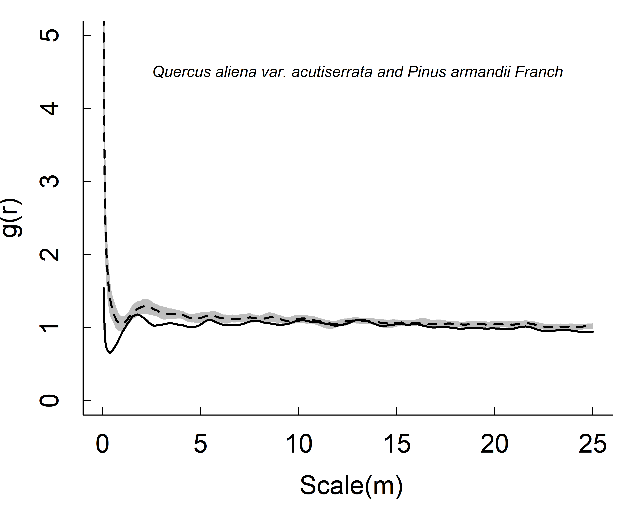

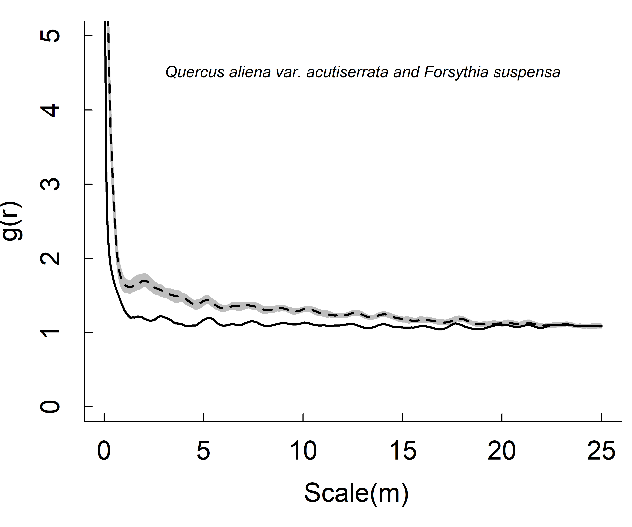


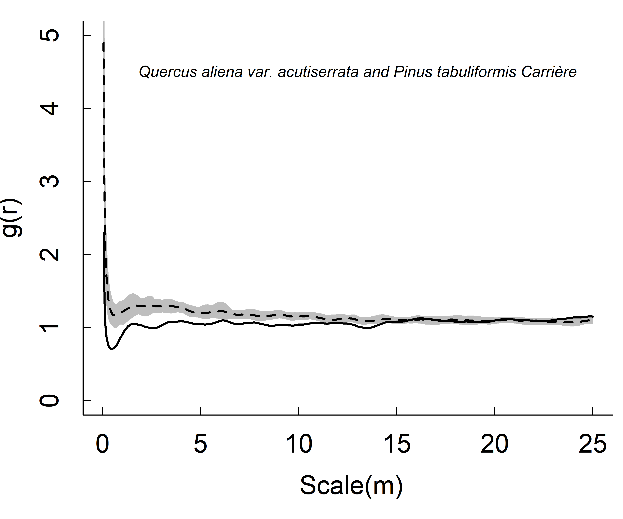

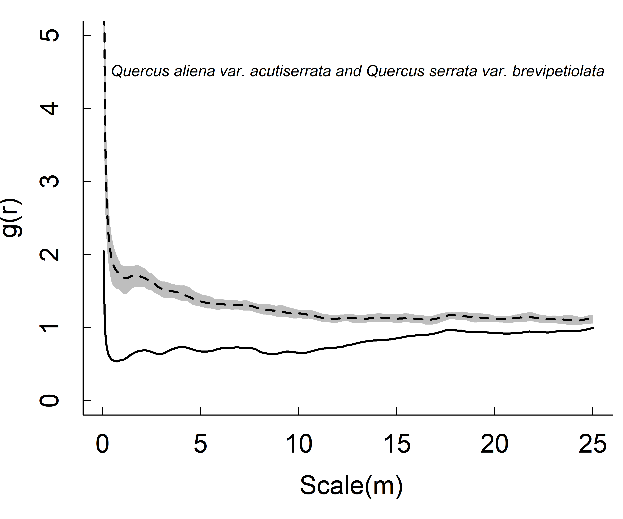


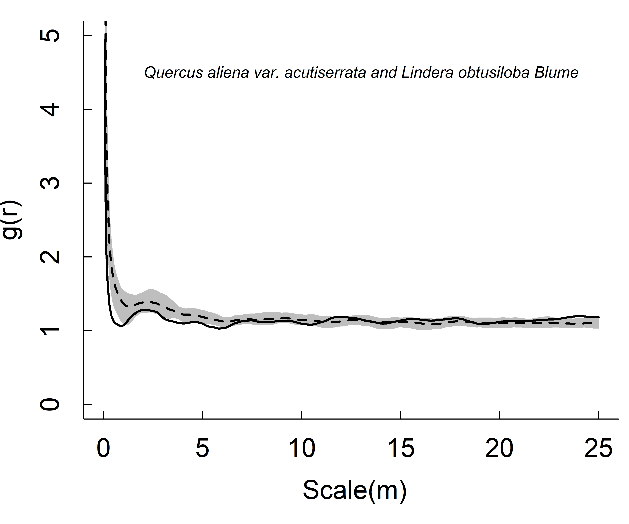

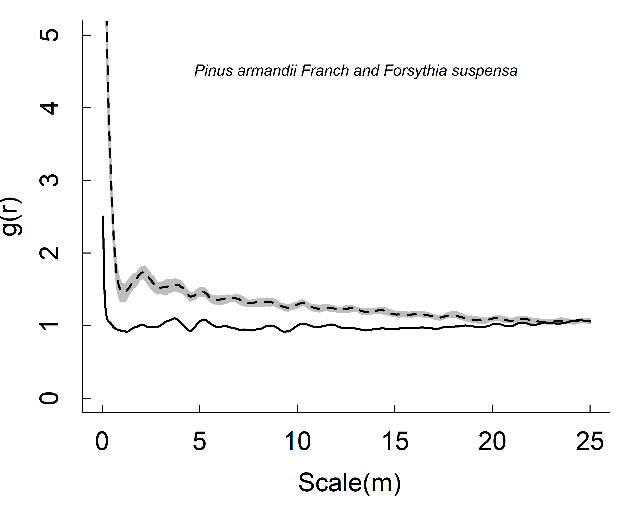


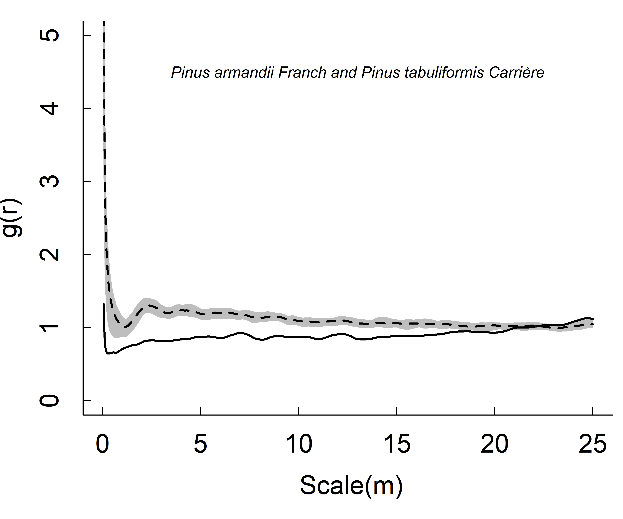

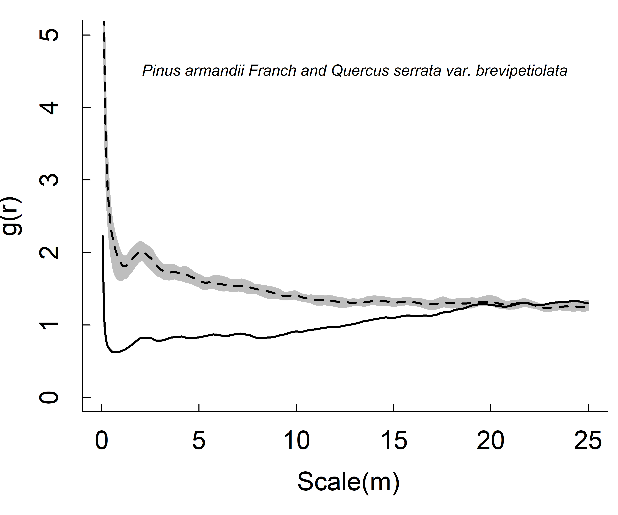


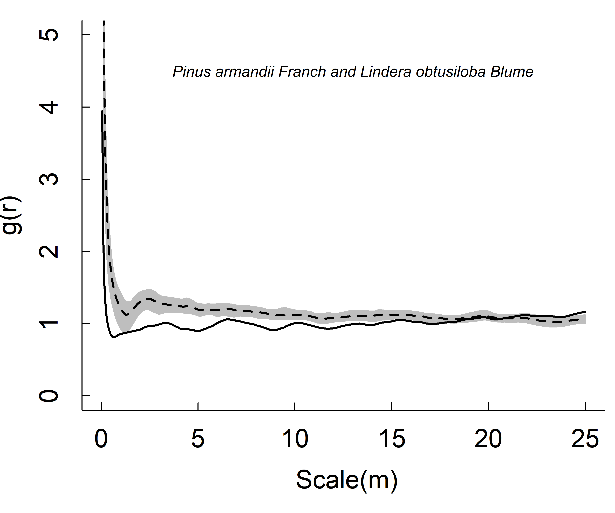

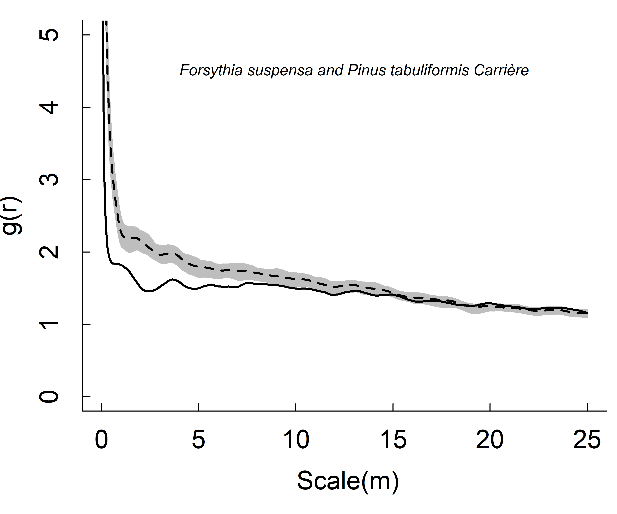


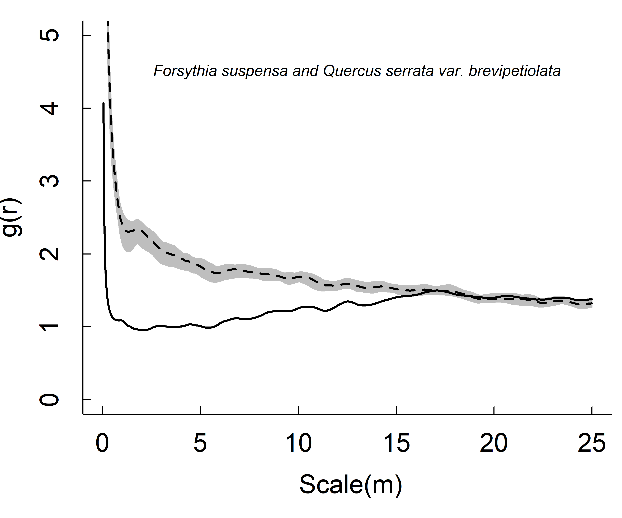

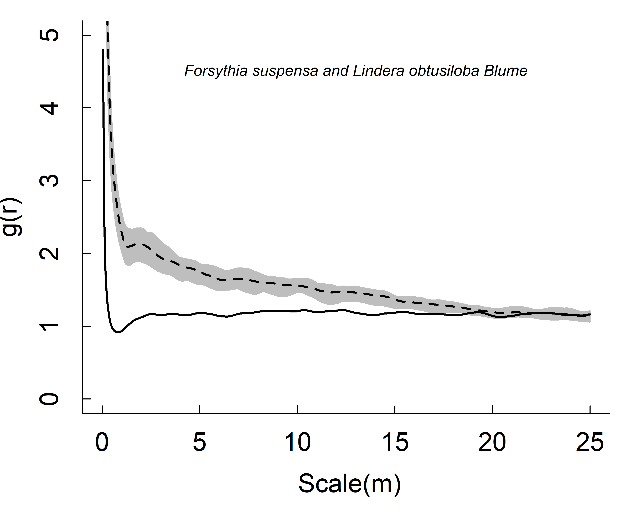


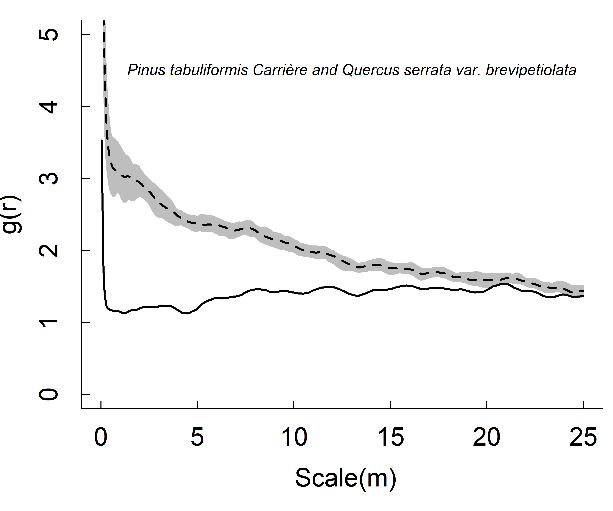

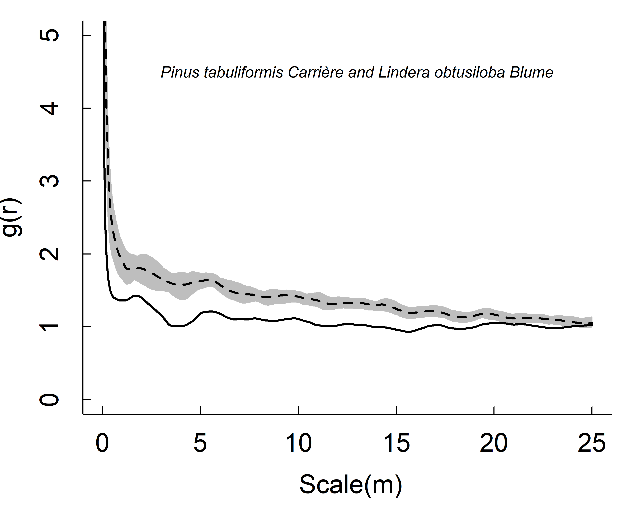


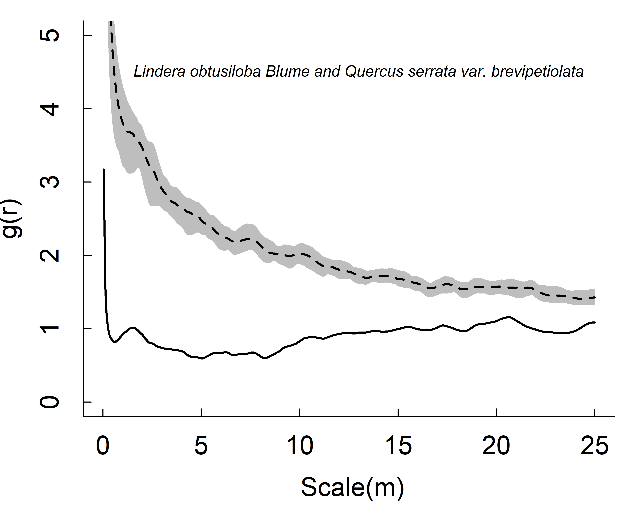


**Figure S4** Analysis of point pattern of 6 dominant species in once-cut forest 1hm^2^ sample plot. The selected function was g(r) function. The solid line denotes the actual interspecific values of the two species, the dashed line denotes the theoretical values, and the gray interval denotes the confidence interval. When g (r) is within the confidence interval, the two species are independent of each other. When g (r) value is above the confidence interval, the two species are significantly positively correlated. When the g (r) value was below the confidence interval, the two species were significantly negatively correlated.


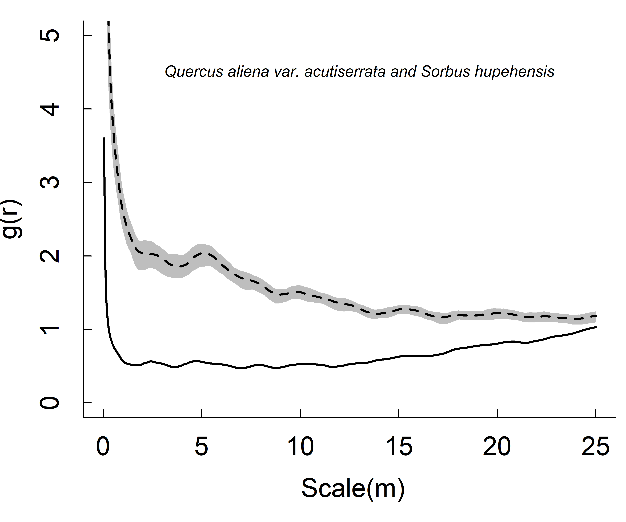

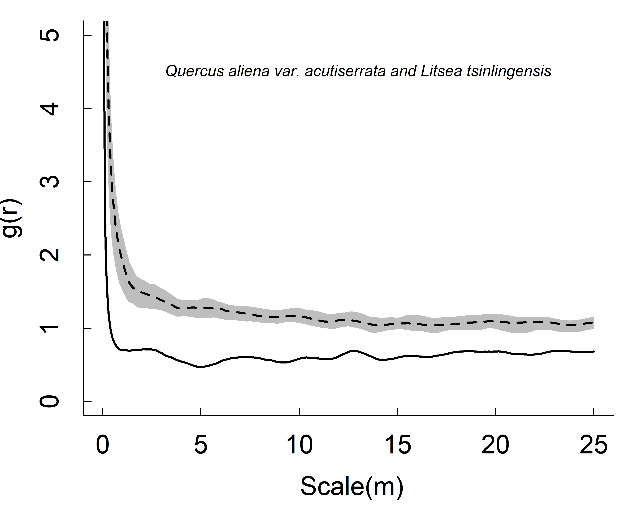


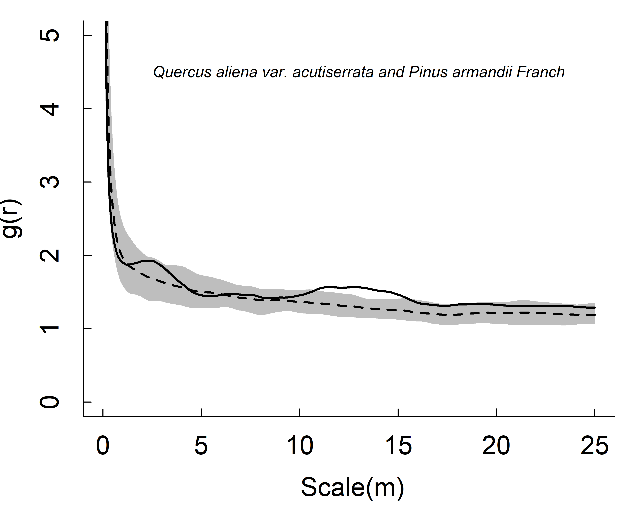

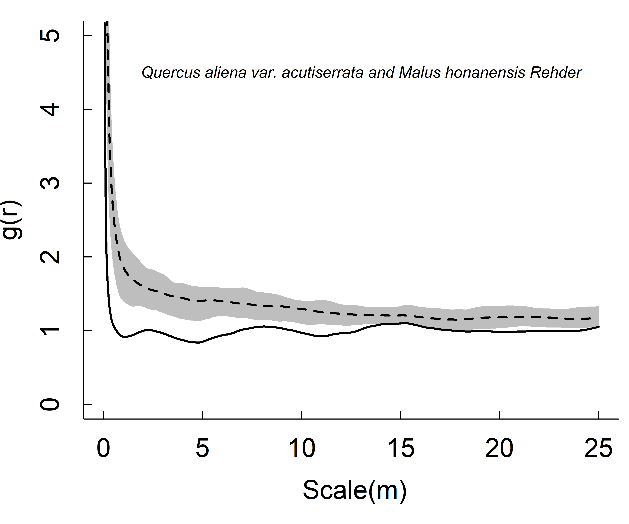


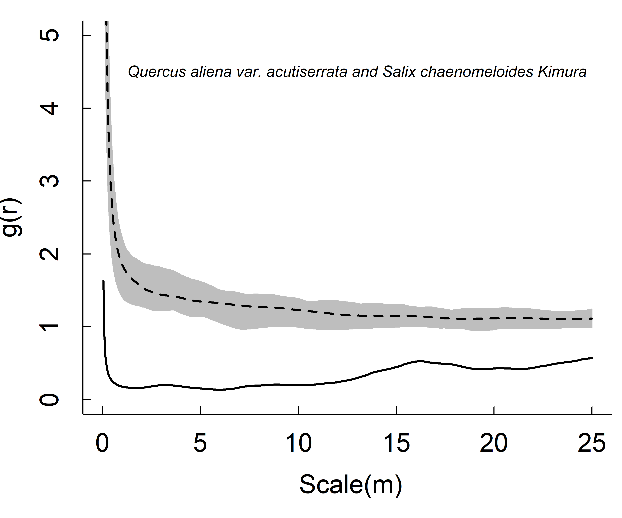

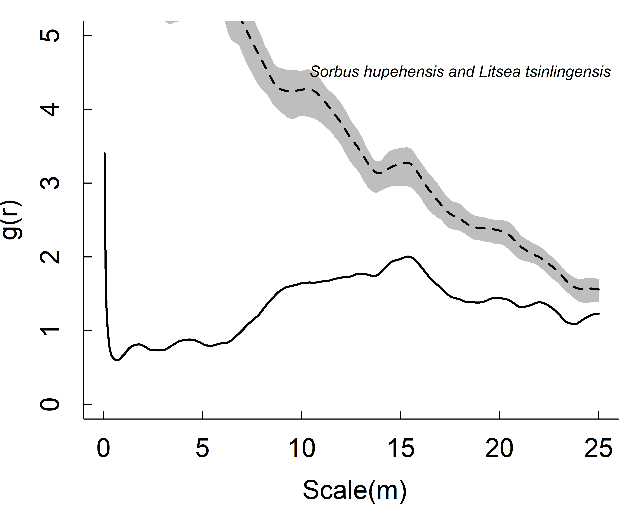


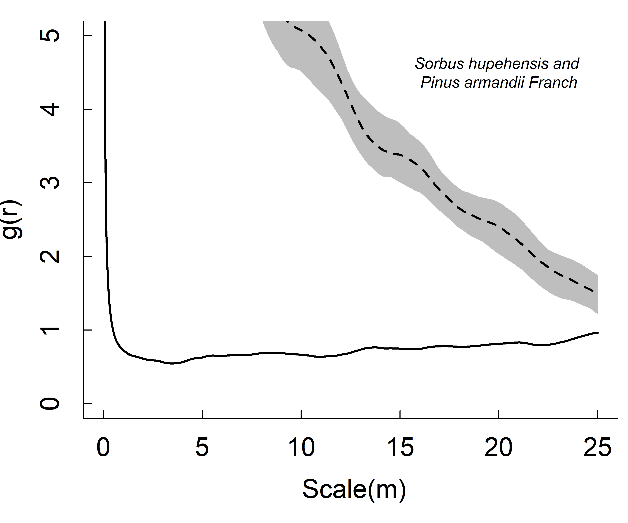

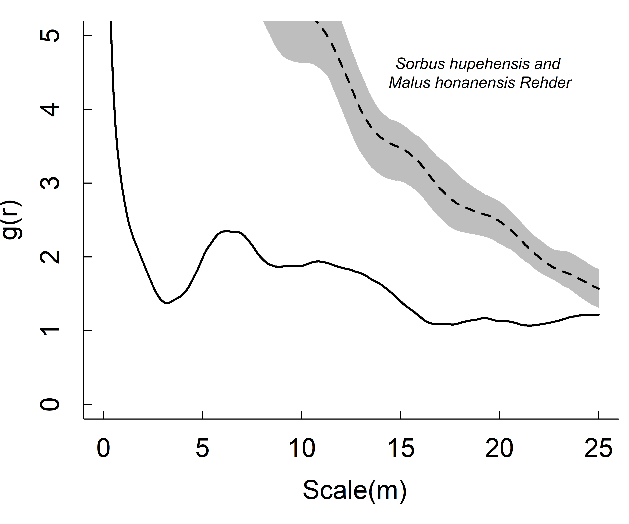


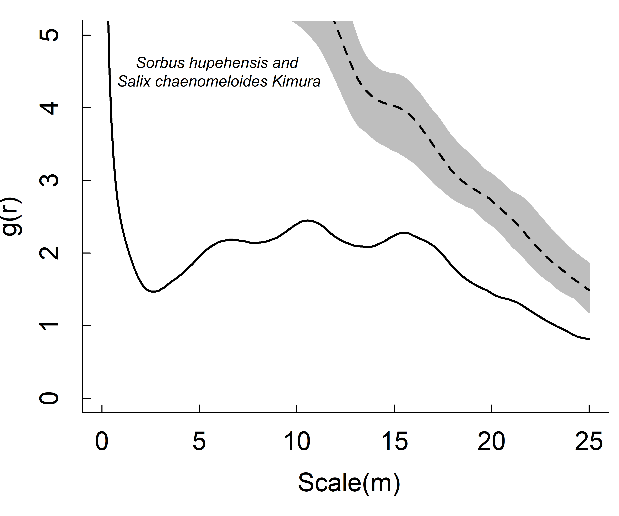

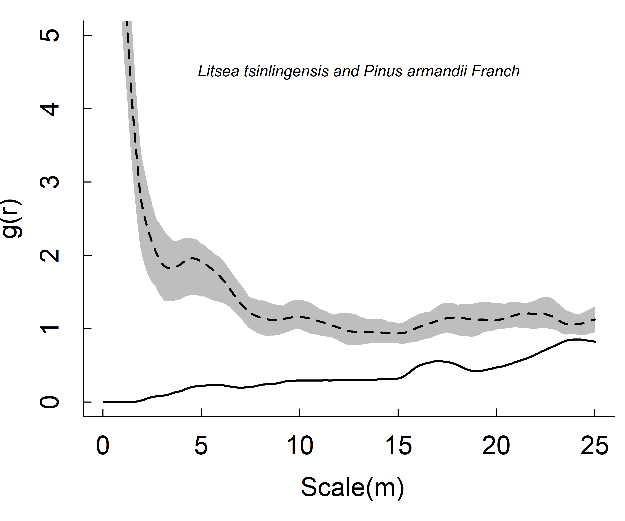


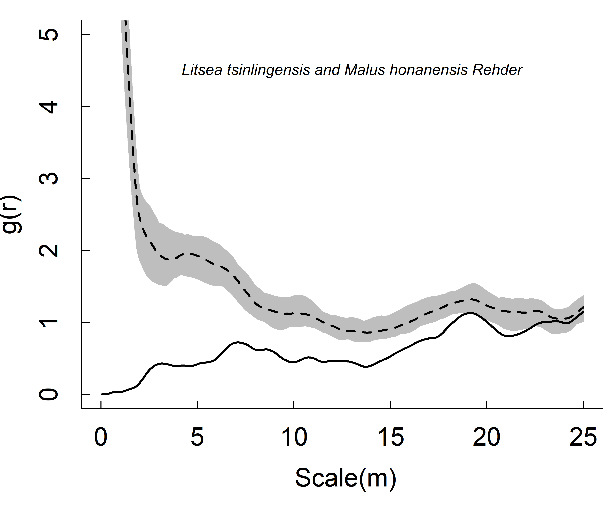

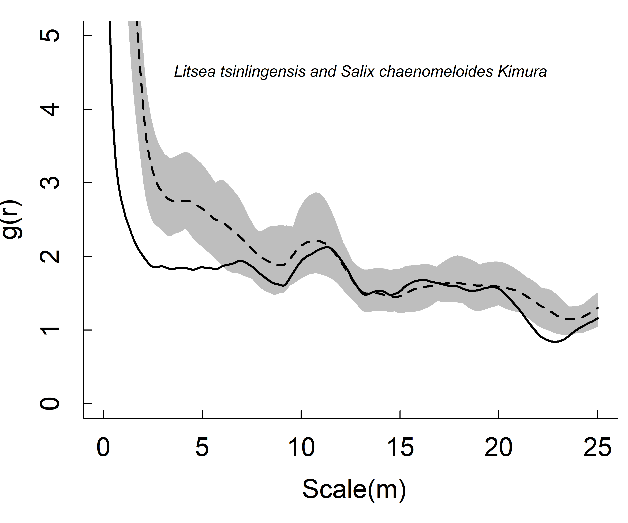


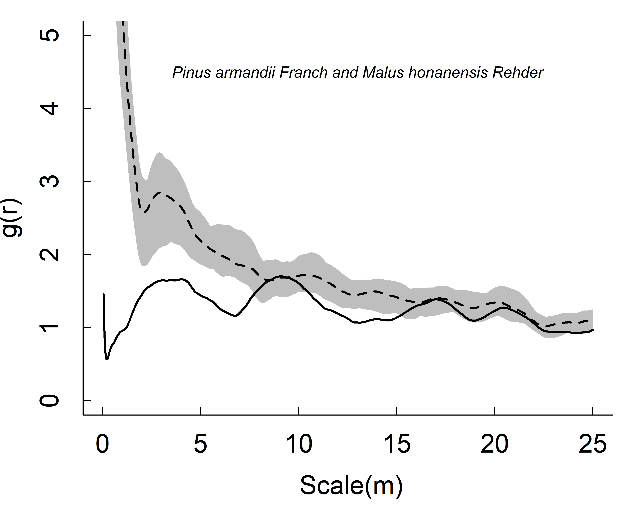

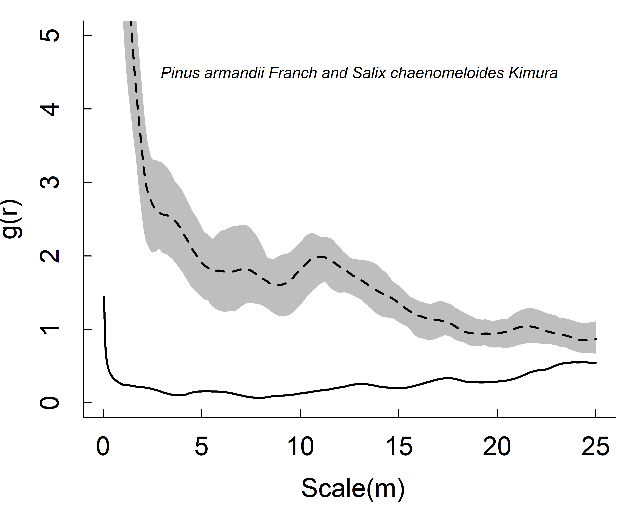


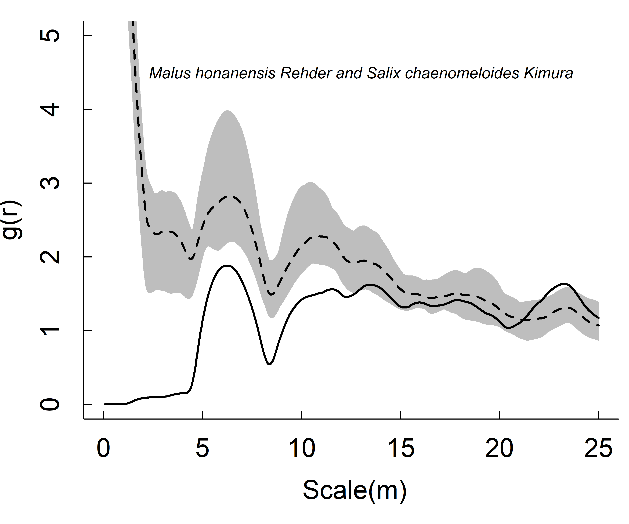


**Figure S5** Analysis of point pattern of 6 dominant species in old growth forest 1hm^2^ sample plot. The selected function was g(r) function. The solid line denotes the actual interspecific values of the two species, the dashed line denotes the theoretical values, and the gray interval denotes the confidence interval. When g (r) is within the confidence interval, the two species are independent of each other. When g (r) value is above the confidence interval, the two species are significantly positively correlated. When the g (r) value was below the confidence interval, the two species were significantly negatively correlated.

**Table S1:** Dominant species in Plantation forest. Important value was calculated as follows: important value = (relative abundance (%) + relative frequency (%) + relative breast height sectional area (%)/3.

| Species | Life form | Abundance | Mean DBH | Bascal area at breast height (cm2) | Importance Value |
| --- | --- | --- | --- | --- | --- |
| *Quercus aliena* var. *acutiserrata* | Tree | 540.00 | 13.09 | 11.30 | 34.13 |
| *Larix gmelinii* | Tree | 125.00 | 24.26 | 6.53 | 14.52 |
| *Pinus tabuliformis Carrière* | Tree | 88.00 | 14.05 | 1.76 | 7.16 |
| *Toxicodendron vernicifluum* | Tree | 76.00 | 12.81 | 1.29 | 6.83 |
| *Juglans cathayensis* | Tree | 45.00 | 21.55 | 2.02 | 6.12 |
| *Cerasus clarofolia* | Shurb | 28.00 | 6.75 | 0.15 | 2.13 |
| *Sorbus alnifolia* | Tree | 25.00 | 6.22 | 0.09 | 2.13 |
| *Cerasus serrulata* | Tree | 25.00 | 7.53 | 0.15 | 1.89 |
| *Litsea tsinlingensis* | Tree | 13.00 | 5.07 | 0.04 | 1.88 |
| *Lindera obtusiloba Blume* | Tree | 14.00 | 5.74 | 0.04 | 1.59 |
| *Crataegus wilsonii* | Shurb | 12.00 | 4.83 | 0.04 | 1.53 |
| *Cornus walteri* | Tree | 14.00 | 12.85 | 0.20 | 1.45 |
| *Betula platyphylla* | Tree | 11.00 | 14.23 | 0.22 | 1.42 |
| *Carpinus cordata* | Tree | 15.00 | 6.60 | 0.07 | 1.33 |
| *Styrax obassis* | Tree | 12.00 | 6.75 | 0.05 | 1.22 |
| *Cornus macrophylla* | Tree | 12.00 | 8.08 | 0.09 | 1.11 |
| *Cornus kousa* subsp. *chinensis* | Shurb | 8.00 | 4.85 | 0.02 | 1.06 |
| *Carpinus turczaninowii* | Tree | 9.00 | 6.31 | 0.04 | 0.95 |
| *Malus honanensis* | Shurb | 7.00 | 3.96 | 0.01 | 0.86 |
| *Pinus armandii Franch* | Tree | 6.00 | 6.28 | 0.02 | 0.85 |
| *Celastrus orbiculatus* | Shurb | 7.00 | 8.66 | 0.12 | 0.84 |
| *Lonicera tatarinowii* | Shurb | 8.00 | 3.84 | 0.01 | 0.73 |
| *Acer pictum* subsp*. mono* | Tree | 7.00 | 6.00 | 0.02 | 0.71 |
| *Malus hupehensis* | Tree | 7.00 | 3.74 | 0.01 | 0.70 |
| *Acer truncatum* | Tree | 6.00 | 5.65 | 0.02 | 0.68 |
| *Meliosma veitchiorum* | Tree | 3.00 | 17.07 | 0.08 | 0.68 |
| *Corylus heterophylla* | Tree | 8.00 | 7.38 | 0.04 | 0.61 |
| *Sorbaria sorbifolia* | Shurb | 2.00 | 20.20 | 0.11 | 0.53 |
| *Acer davidii* | Tree | 5.00 | 7.32 | 0.03 | 0.51 |
| *Acer davidii* subsp. *grosseri* | Tree | 4.00 | 6.65 | 0.02 | 0.46 |
| *Symplocos paniculata* | Shurb | 4.00 | 3.05 | 0.00 | 0.44 |
| *Padus buergeriana* | Tree | 2.00 | 7.60 | 0.01 | 0.39 |
| *Tilia japonica* | Tree | 2.00 | 6.10 | 0.01 | 0.39 |
| *Betula chinensis* | Tree | 3.00 | 6.60 | 0.01 | 0.26 |
| *Padus obtusata* | Tree | 2.00 | 12.95 | 0.03 | 0.26 |
| *Viburnum lobophyllum* | Shurb | 3.00 | 2.43 | 0.00 | 0.25 |
| *Rosa bella* | Shurb | 2.00 | 1.20 | 0.00 | 0.22 |
| *Salix tangii* | Shurb | 1.00 | 12.40 | 0.01 | 0.21 |
| *Yulania denudata* | Tree | 1.00 | 9.60 | 0.01 | 0.20 |
| *Meliosma flexuosa* | Tree | 1.00 | 5.30 | 0.00 | 0.19 |
| *Crataegus hupehensis* | Tree | 1.00 | 4.40 | 0.00 | 0.19 |
| *Tilia paucicostata* | Tree | 1.00 | 2.90 | 0.00 | 0.19 |

**Table S2:** Dominant species in twice-cut forest. Important value was calculated as follows: important value = (relative abundance (%) + relative frequency (%) + relative breast height sectional area (%)/3.

| Species | Life form | Abundance | Mean DBH | Bascal area at breast height (cm2) | Importance Value |
| --- | --- | --- | --- | --- | --- |
| *Quercus aliena* var. *acutiserrata* | Tree | 1002.00 | 10.02 | 16.30 | 34.07 |
| *Pinus armandii Franch* | Tree | 203.00 | 14.46 | 4.01 | 9.21 |
| *Corylus heterophylla* | Tree | 456.00 | 3.03 | 0.46 | 7.66 |
| *Toxicodendron vernicifluum* | Tree | 159.00 | 9.50 | 1.68 | 6.27 |
| *Lindera obtusiloba Blume* | Tree | 170.00 | 6.07 | 0.59 | 5.03 |
| *Betula platyphylla* | Tree | 54.00 | 11.22 | 0.86 | 3.95 |
| *Sorbus alnifolia* | Tree | 130.00 | 4.96 | 0.37 | 3.85 |
| *Cornus kousa* subsp. *chinensis* | Shurb | 134.00 | 5.11 | 0.36 | 3.73 |
| *Symplocos paniculata* | Shurb | 150.00 | 2.86 | 0.11 | 3.14 |
| *Malus honanensis* | Shurb | 95.00 | 3.65 | 0.17 | 2.92 |
| *Forsythia suspensa* | Shurb | 181.00 | 2.03 | 0.07 | 2.66 |
| *Litsea tsinlingensis* | Tree | 51.00 | 4.17 | 0.08 | 2.18 |
| *Carpinus turczaninowii* | Tree | 25.00 | 5.35 | 0.07 | 1.58 |
| *Rhododendron micranthum* | Shurb | 51.00 | 2.44 | 0.03 | 1.20 |
| *Philadelphus incanus* | Shurb | 33.00 | 2.18 | 0.01 | 1.14 |
| *Tilia japonica* | Tree | 13.00 | 9.58 | 0.19 | 1.14 |
| *Carpinus cordata* | Tree | 13.00 | 4.68 | 0.03 | 0.94 |
| *Cerasus clarofolia* | Shurb | 11.00 | 9.43 | 0.15 | 0.92 |
| *Meliosma veitchiorum* | Tree | 12.00 | 8.62 | 0.13 | 0.75 |
| *Acer davidii* | Tree | 11.00 | 8.94 | 0.08 | 0.68 |
| *Viburnum betulifolium* | Shurb | 17.00 | 2.78 | 0.01 | 0.66 |
| *Celastrus orbiculatus* | Shurb | 11.00 | 3.36 | 0.01 | 0.59 |
| *Pinus tabuliformis Carrière* | Tree | 12.00 | 10.20 | 0.11 | 0.58 |
| *Cornus controversa* | Tree | 5.00 | 4.66 | 0.01 | 0.52 |
| *Quercus variabilis* | Tree | 2.00 | 36.05 | 0.20 | 0.43 |
| *Tilia paucicostata* | Tree | 3.00 | 12.27 | 0.05 | 0.39 |
| *Betula luminifera* | Tree | 5.00 | 4.64 | 0.01 | 0.37 |
| *Quercus serrata* var. *brevipetiolata* | Tree | 3.00 | 3.53 | 0.00 | 0.34 |
| *Juglans cathayensis* | Tree | 3.00 | 17.30 | 0.11 | 0.32 |
| *Betula chinensis* | Tree | 5.00 | 8.66 | 0.06 | 0.28 |
| *Berberis circumserrata* | Shurb | 3.00 | 12.10 | 0.07 | 0.28 |
| *Yulania denudata* | Tree | 1.00 | 29.60 | 0.07 | 0.25 |
| *Sorbus hupehensis* | Shurb | 8.00 | 2.96 | 0.01 | 0.25 |
| *Corylus chinensis* | Tree | 8.00 | 2.99 | 0.01 | 0.25 |
| *Populus davidiana* | Tree | 1.00 | 23.60 | 0.04 | 0.22 |
| *Cotoneaster acutifolius* | Shurb | 5.00 | 2.06 | 0.00 | 0.21 |
| *Cornus macrophylla* | Tree | 3.00 | 4.60 | 0.01 | 0.19 |
| *Meliosma flexuosa* | Tree | 1.00 | 12.60 | 0.01 | 0.18 |
| *Abelia uniflora* | Shurb | 2.00 | 2.80 | 0.00 | 0.18 |
| *Cornus schindleri* subsp. *poliophylla* | Tree | 1.00 | 5.00 | 0.00 | 0.17 |
| *Elaeagnus umbellata* | Shurb | 1.00 | 1.80 | 0.00 | 0.16 |
| *Crataegus pinnatifida* | Tree | 4.00 | 10.28 | 0.04 | 0.10 |
| *Styrax obassis* | Tree | 1.00 | 13.20 | 0.01 | 0.03 |
| *Elaeagnus angustifolia* | Tree | 2.00 | 3.50 | 0.00 | 0.02 |
| *Acer truncatum* | Tree | 2.00 | 2.45 | 0.00 | 0.02 |
| *Sorbaria sorbifolia* | Shurb | 2.00 | 2.30 | 0.00 | 0.02 |

**Table S3:** Dominant species in once-cut forest. Important value was calculated as follows: important value = (relative abundance (%) + relative frequency (%) + relative breast height sectional area (%)/3.

| Species | Life form | Abundance | Mean DBH | Bascal area at breast height (cm2) | Importance Value |
| --- | --- | --- | --- | --- | --- |
| *Quercus aliena* var. *acutiserrata* | Tree | 752.00 | 14.30 | 14.90 | 21.49 |
| *Pinus armandii Franch* | Tree | 574.00 | 11.35 | 6.63 | 12.54 |
| *Forsythia suspensa* | Shurb | 986.00 | 2.19 | 0.69 | 10.29 |
| *Pinus tabuliformis Carrière* | Tree | 343.00 | 12.57 | 5.12 | 9.29 |
| *Quercus serrata* var*. brevipetiolata* | Tree | 379.00 | 8.59 | 3.67 | 7.59 |
| *Lindera obtusiloba Blume* | Tree | 246.00 | 4.55 | 0.55 | 4.35 |
| *Toxicodendron vernicifluum* | Tree | 111.00 | 12.12 | 1.48 | 3.75 |
| *Sorbus alnifolia* | Tree | 169.00 | 2.94 | 0.16 | 3.16 |
| *Symplocos paniculata* | Shurb | 113.00 | 2.50 | 0.11 | 2.60 |
| *Carpinus turczaninowii* | Tree | 76.00 | 4.14 | 0.17 | 2.52 |
| *Litsea tsinlingensis* | Tree | 95.00 | 4.26 | 0.32 | 2.40 |
| *Betula platyphylla* | Tree | 59.00 | 7.92 | 0.38 | 2.34 |
| *Acer davidii* | Tree | 26.00 | 11.09 | 0.46 | 1.43 |
| *Tilia paucicostata* | Tree | 34.00 | 3.31 | 0.04 | 1.19 |
| *Tilia japonica* | Tree | 54.00 | 2.95 | 0.05 | 1.19 |
| *Cotoneaster acutifolius* | Shurb | 43.00 | 1.80 | 0.03 | 1.17 |
| *Cerasus clarofolia* | Shurb | 25.00 | 9.76 | 0.27 | 1.00 |
| *Juglans cathayensis* | Tree | 12.00 | 14.94 | 0.29 | 0.92 |
| *Philadelphus incanus* | Shurb | 26.00 | 1.68 | 0.01 | 0.85 |
| *Carpinus cordata* | Tree | 22.00 | 6.47 | 0.11 | 0.83 |
| *Cornus kousa* subsp. *chinensis* | Shurb | 20.00 | 3.11 | 0.02 | 0.82 |
| *Meliosma veitchiorum* | Tree | 9.00 | 10.43 | 0.10 | 0.73 |
| *Larix gmelinii* | Tree | 8.00 | 21.98 | 0.38 | 0.65 |
| *Crataegus wilsonii* | Shurb | 8.00 | 1.89 | 0.00 | 0.63 |
| *Cornus controversa* | Tree | 11.00 | 7.58 | 0.08 | 0.48 |
| *Corylus heterophylla* | Tree | 10.00 | 2.11 | 0.00 | 0.40 |
| *Yulania denudata* | Tree | 5.00 | 7.08 | 0.03 | 0.39 |
| *Cornus macrophylla* | Tree | 8.00 | 8.30 | 0.06 | 0.36 |
| *Cerasus serrulata* | Tree | 3.00 | 11.23 | 0.05 | 0.31 |
| *Quercus variabilis* | Tree | 7.00 | 10.77 | 0.09 | 0.30 |
| *Malus honanensis* | Shurb | 6.00 | 1.77 | 0.00 | 0.29 |
| *Corylus chinensis* | Tree | 4.00 | 2.20 | 0.00 | 0.28 |
| *Acer pictum* subsp. *mono* | Tree | 3.00 | 3.23 | 0.00 | 0.27 |
| *Carpinus polyneura* | Tree | 4.00 | 6.95 | 0.02 | 0.21 |
| *Acer truncatum* | Tree | 5.00 | 5.18 | 0.01 | 0.21 |
| *Cornus walteri* | Tree | 3.00 | 7.57 | 0.02 | 0.20 |
| *Maackia hwashanensis* | Tree | 4.00 | 2.98 | 0.00 | 0.20 |
| *Aralia elata* | Shurb | 2.00 | 8.15 | 0.01 | 0.19 |
| *Malus hupehensis* | Tree | 2.00 | 3.75 | 0.00 | 0.18 |
| *Fraxinus paxiana* | Tree | 2.00 | 3.35 | 0.00 | 0.18 |
| *Celtis koraiensis* | Tree | 2.00 | 2.90 | 0.00 | 0.18 |
| *Carpinus viminea* | Tree | 3.00 | 12.70 | 0.04 | 0.14 |
| *Euptelea pleiosperma* | Tree | 3.00 | 6.77 | 0.01 | 0.12 |
| *Rhododendron micranthum* | Shurb | 4.00 | 2.00 | 0.00 | 0.11 |
| *Cornus hemsleyi* | Tree | 1.00 | 16.80 | 0.02 | 0.11 |
| *Celastrus orbiculatus* | Shurb | 3.00 | 4.47 | 0.00 | 0.11 |
| *Ostrya japonica* | Tree | 2.00 | 9.00 | 0.01 | 0.11 |
| *Ulmus davidiana* | Tree | 3.00 | 1.90 | 0.00 | 0.11 |
| *Fraxinus chinensis* | Tree | 2.00 | 5.20 | 0.00 | 0.10 |
| *Acer davidii* subsp. *grosseri* | Tree | 2.00 | 2.65 | 0.00 | 0.10 |
| *Euonymus phellomanus* | Shurb | 2.00 | 2.85 | 0.00 | 0.10 |
| *Meliosma flexuosa* | Tree | 1.00 | 6.00 | 0.00 | 0.09 |
| *Betula luminifera* | Tree | 1.00 | 5.40 | 0.00 | 0.09 |
| *Styrax obassis* | Tree | 1.00 | 3.80 | 0.00 | 0.09 |
| *Fraxinus bungeana* | Tree | 1.00 | 2.70 | 0.00 | 0.09 |
| *Viburnum betulifolium* | Shurb | 1.00 | 1.50 | 0.00 | 0.09 |
| *Lonicera microphylla* | Shurb | 1.00 | 1.00 | 0.00 | 0.09 |

**Table S4:** Dominant species in old-growth forest. Important value was calculated as follows: important value = (relative abundance (%) + relative frequency (%) + relative breast height sectional area (%)/3.

| Species | Life form | Abundance | Mean DBH | Bascal area at breast height (cm2) | Importance Value |
| --- | --- | --- | --- | --- | --- |
| *Quercus aliena* var*. acutiserrata* | Tree | 938.00 | 16.32 | 26.39 | 42.35 |
| *Sorbus hupehensis* | Shurb | 382.00 | 2.71 | 0.36 | 6.59 |
| *Litsea tsinlingensis* | Tree | 183.00 | 4.68 | 0.50 | 5.05 |
| *Pinus armandii Franch* | Tree | 76.00 | 10.04 | 1.00 | 3.88 |
| *Malus honanensis* | Shurb | 104.00 | 5.09 | 0.42 | 3.42 |
| *Salix chaenomeloides Kimura* | Tree | 84.00 | 10.56 | 0.94 | 2.94 |
| *Pinus tabuliformis Carrière* | Tree | 32.00 | 10.63 | 0.55 | 2.46 |
| *Sorbus alnifolia* | Tree | 36.00 | 5.64 | 0.11 | 2.44 |
| *Lindera obtusiloba Blume* | Tree | 43.00 | 5.29 | 0.15 | 2.33 |
| *Cerasus clarofolia* | Shurb | 67.00 | 6.73 | 0.33 | 2.10 |
| *Cotoneaster acutifolius* | Shurb | 60.00 | 2.72 | 0.09 | 2.00 |
| *Tilia japonica* | Tree | 31.00 | 6.08 | 0.16 | 1.94 |
| *Fraxinus chinensis* | Tree | 43.00 | 8.20 | 0.34 | 1.78 |
| *Euonymus phellomanus* | Shurb | 60.00 | 2.56 | 0.05 | 1.72 |
| *Acer pictum* subsp. *mono* | Tree | 26.00 | 4.08 | 0.05 | 1.63 |
| *Abelia biflora* | Shurb | 50.00 | 2.16 | 0.02 | 1.55 |
| *Toxicodendron vernicifluum* | Tree | 17.00 | 13.14 | 0.32 | 1.30 |
| *Populus davidiana* | Tree | 27.00 | 6.11 | 0.14 | 1.24 |
| *Philadelphus incanus* | Shurb | 43.00 | 2.67 | 0.03 | 1.22 |
| *Forsythia suspensa* | Shurb | 29.00 | 2.39 | 0.02 | 1.15 |
| *Celastrus orbiculatus* | Shurb | 17.00 | 4.44 | 0.03 | 1.12 |
| *Cornus macrophylla* | Tree | 20.00 | 6.61 | 0.10 | 0.86 |
| *Ailanthus altissima* | Tree | 8.00 | 3.96 | 0.01 | 0.86 |
| *Salix shihtsuanensis* | Shurb | 16.00 | 6.86 | 0.08 | 0.67 |
| *Corylus chinensis* | Tree | 10.00 | 15.88 | 0.23 | 0.62 |
| *Sambucus williamsii* | Shurb | 9.00 | 2.93 | 0.01 | 0.50 |
| *Acer davidii* | Tree | 7.00 | 6.80 | 0.04 | 0.50 |
| *Alangium platanifolium* | Shurb | 8.00 | 2.36 | 0.00 | 0.48 |
| *Lonicera tatarinowii* | Shurb | 8.00 | 1.79 | 0.00 | 0.48 |
| *Corylus heterophylla* | Tree | 5.00 | 4.98 | 0.01 | 0.45 |
| *Crataegus wilsonii* | Shurb | 5.00 | 3.02 | 0.00 | 0.44 |
| *Carpinus cordata* | Tree | 3.00 | 9.97 | 0.03 | 0.32 |
| *Berberis circumserrata* | Shurb | 5.00 | 1.40 | 0.00 | 0.31 |
| *Symplocos paniculata* | Shurb | 2.00 | 3.65 | 0.00 | 0.28 |
| *Rosa bella* | Shurb | 2.00 | 1.70 | 0.00 | 0.27 |
| *Juglans cathayensis* | Tree | 3.00 | 20.20 | 0.10 | 0.27 |
| *Quercus serrata* var. *brevipetiolata* | Tree | 4.00 | 13.05 | 0.06 | 0.24 |
| *Viburnum opulus* var. *sargentii* | Shurb | 7.00 | 3.23 | 0.01 | 0.22 |
| *Populus simonii* | Tree | 5.00 | 2.04 | 0.00 | 0.19 |
| *Clematis heracleifolia* | Shurb | 2.00 | 4.25 | 0.00 | 0.15 |
| *Viburnum sargentii* f*. calvescens* | Shurb | 2.00 | 2.40 | 0.00 | 0.15 |
| *Betula luminifera* | Tree | 1.00 | 12.20 | 0.01 | 0.15 |
| *Padus buergeriana* | Tree | 1.00 | 9.60 | 0.01 | 0.14 |
| *Acer truncatum* | Tree | 1.00 | 9.00 | 0.01 | 0.14 |
| *Carpinus turczaninowii* | Tree | 1.00 | 8.70 | 0.01 | 0.14 |
| *Padus velutina* | Tree | 1.00 | 7.00 | 0.00 | 0.14 |
| *Betula albosinensis* | Tree | 1.00 | 6.30 | 0.00 | 0.14 |
| *Fraxinus chinensis* subsp*. Rhynchophylla* | Tree | 1.00 | 6.00 | 0.00 | 0.14 |
| *Evodia daniellii* | Tree | 1.00 | 5.10 | 0.00 | 0.14 |
| *Cotoneaster tenuipes* | Shurb | 1.00 | 4.70 | 0.00 | 0.14 |
| *Meliosma veitchiorum* | Tree | 1.00 | 2.50 | 0.00 | 0.14 |
| *Euptelea pleiosperma* | Tree | 1.00 | 1.00 | 0.00 | 0.14 |
